# Deconvolution of monocyte responses in inflammatory bowel disease reveals an IL-1 cytokine network that regulates IL-23 in genetic and acquired IL-10 resistance

**DOI:** 10.1136/gutjnl-2020-321731

**Published:** 2020-10-09

**Authors:** Dominik Aschenbrenner, Maria Quaranta, Soumya Banerjee, Nicholas Ilott, Joanneke Jansen, Boyd Steere, Yin-Huai Chen, Stephen Ho, Karen Cox, Carolina V Arancibia-Cárcamo, Mark Coles, Eamonn Gaffney, Simon PL Travis, Lee Denson, Subra Kugathasan, Jochen Schmitz, Fiona Powrie, Stephen N Sansom, Holm H Uhlig

**Affiliations:** 1 Translational Gastroenterology Unit, NIHR Oxford Biomedical Research Centre, John Radcliffe Hospital, University of Oxford, Oxford, Oxfordshire, UK; 2 IBD Center, Laboratory of Gastrointestinal Immunopathology, Humanitas Clinical and Research Center, Milan, Italy; 3 Department of Psychology, University of Cambridge, Cambridge, Cambridgeshire, UK; 4 Kennedy Institute of Rheumatology, University of Oxford, Oxford, Oxfordshire, UK; 5 Wolfson Centre for Mathematical Biology, University of Oxford, Oxford, Oxfordshire, UK; 6 Immunology Translational Sciences, Eli Lilly and Company, Indianapolis, Indiana, USA; 7 Pediatric Gastroenterology, Cincinnati Childrens Hospital Medical Center, Cincinnati, Ohio, USA; 8 Pediatrics, Emory University School of Medicine, Atlanta, Georgia, USA; 9 Department of Paediatrics, University of Oxford, Oxford, Oxfordshire, UK

**Keywords:** mucosal immunology, inflammatory bowel disease, interleukins

## Abstract

**Objective:**

Dysregulated immune responses are the cause of IBDs. Studies in mice and humans suggest a central role of interleukin (IL)-23-producing mononuclear phagocytes in disease pathogenesis. Mechanistic insights into the regulation of IL-23 are prerequisite for selective IL-23 targeting therapies as part of personalised medicine.

**Design:**

We performed transcriptomic analysis to investigate IL-23 expression in human mononuclear phagocytes and peripheral blood mononuclear cells. We investigated the regulation of IL-23 expression and used single-cell RNA sequencing to derive a transcriptomic signature of hyperinflammatory monocytes. Using gene network correlation analysis, we deconvolved this signature into components associated with homeostasis and inflammation in patient biopsy samples.

**Results:**

We characterised monocyte subsets of healthy individuals and patients with IBD that express IL-23. We identified autosensing and paracrine sensing of IL-1α/IL-1β and IL-10 as key cytokines that control IL-23-producing monocytes. Whereas Mendelian genetic defects in IL-10 receptor signalling induced IL-23 secretion after lipopolysaccharide stimulation, whole bacteria exposure induced IL-23 production in controls via acquired IL-10 signalling resistance. We found a transcriptional signature of IL-23-producing inflammatory monocytes that predicted both disease and resistance to antitumour necrosis factor (TNF) therapy and differentiated that from an IL-23-associated lymphocyte differentiation signature that was present in homeostasis and in disease.

**Conclusion:**

Our work identifies IL-10 and IL-1 as critical regulators of monocyte IL-23 production. We differentiate homeostatic IL-23 production from hyperinflammation-associated IL-23 production in patients with severe ulcerating active Crohn’s disease and anti-TNF treatment non-responsiveness. Altogether, we identify subgroups of patients with IBD that might benefit from IL-23p19 and/or IL-1α/IL-1β-targeting therapies upstream of IL-23.

**Significance of this study**

**What is already known on this subject?**

- IBD is a complex chronic inflammatory disease caused by the interaction of host and environmental factors in a genetically susceptible setting. Targeting the proinflammatory arm of the immune system such as by blocking tumour necrosis factor (TNF) signalling has proven therapeutically successful in subgroups of patients with IBD. Studies in both mice and humans indicate interleukin (IL)-23 as a key pathogenic proinflammatory factor in IBD. Early-stage clinical trials indicate that blocking IL-23 can induce disease remission in patients with Crohn’s disease (CD) and ulcerative colitis (UC). As part of personalised medicine, mechanistic insights into the regulation of IL-23 are prerequisite for selective IL-23 targeting therapies.

**What are the new findings?**

- We identify autocrine and paracrine sensing of IL-1α/IL-1β and IL-10 as key cytokines that control IL-23-producing monocytes. We determine a transcriptional signature of IL-23-producing monocytes that predicted both disease and resistance to anti-TNF therapy and differentiate this hyperinflammation-associated IL-23 production in patients with severe ulcerating active CD from homeostatic intestinal IL-23 production. We show that exposure of monocytes to whole bacteria induced IL-23 production via acquired IL-10 signalling resistance akin to IL-23 secretion by lipopolysaccharide-stimulated monocytes in Mendelian IL-10 signalling defects.

**Significance of this study**

**How might it impact on clinical practice in the foreseeable future?**

- We identify subgroups of patients with IBD that might benefit from IL-23p19 and/or IL-1α/IL-1β-targeting therapies upstream of IL-23.

## Introduction

The pathogenesis of inflammatory bowel disease (IBD), which includes Crohn’s disease (CD) and ulcerative colitis (UC) and IBD unclassified (IBDu), is caused by dysregulated innate and adaptive immune responses that drive chronic relapsing tissue inflammation.^1^ Genetic studies suggest a complex polygenic inheritance driven by defective innate and adaptive immunity.^1 2^ Interleukin (IL)-23 signalling and T-helper (Th)1/Th17 immunity are significant mediators of intestinal inflammation as indicated by genetic variants in *IL23R* encoding the IL-23 receptor^3^ as well as *RORC*, *STAT3*, *IRF5*, *IL1R1*, *IL6ST*, *IL12B, TYK2, IL21, JAK2, IFNG, SMAD3* and *CCR6*.^4 5^ Monocytes, macrophages and dendritic cells (DCs) produce IL-23 in response to bacteria- and fungi-derived microbial stimuli and drive Th1 and Th17 differentiation in the pathogenesis of intestinal inflammation.^6–9^ In addition to a role of pro-inflammatory and anti-inflammatory cytokine networks,^10^ mouse models as well as human Mendelian disorders highlight the essential role of IL-10 signalling in controlling inflammatory cytokine responses. Therapeutic approaches that target the pro-inflammatory arm of the immune system by blocking cytokine signalling or by affecting intestinal cell migration are effective in subgroups of patients with IBD.^8^ Mice that lack IL-23p19 are protected from developing colitis in innate as well as lymphocyte replete models of intestinal inflammation induced by IL-10 signalling defects, bacterial colonisation or innate immune stimulation *via* the anti-CD40.^7 11^ These results indicate that IL-10 blockade unleashes an IL-23 mediated immune pathogenesis. Importantly, blockade of IL-23p19 and IL-12p40 showed therapeutic benefit for patients with CD and UC.^12 13^

To predict genetic susceptibility or disease course, individual loci and genetic risk scores^14 15^ as well as gene expression signatures or transcriptomic scores of peripheral CD8^+^ T cells,^16^ peripheral blood^17^ or intestinal tissue^18^ are revealing patient stratification strategies in IBD. Membrane-bound tumour necrosis factor (TNF)^19^ and IL-6/oncostatin M (OSM) associated cytokines^18^ predict anti-TNF non-response. However, transcriptional signatures informed by IL-23 expression have not been described.

Personalised medicine targeting the IL-23 axis requires an understanding of the cellular sources, networks and regulation of IL-23. Here we investigate the regulation of IL-23, describe distinct monocyte subsets that express IL-23 and identify IL-1 signalling as the key cytokine for the differentiation of IL-23-producing monocytes. We identify a hyperinflammatory signature of IL-23-producing monocytes in intestinal tissue transcriptomes of patients with IBD and find an additional signature of IL-23 that is associated with lymphocyte cell differentiation in healthy tissue.

## Methods

Detailed methods are outlined in the online supplemental materials.

10.1136/gutjnl-2020-321731.supp1 Supplementary data

## Results

### IL-10 signalling blockade facilitates IL-23 production by a subset of peripheral blood monocytes

We investigated the regulation of *IL23A* expression by subjecting peripheral blood mononuclear cells (PBMC) from 41 patients with IBD (online supplemental figure 1A and online supplemental table 1) to IBD-relevant stimuli that target different aspects of innate and adaptive immune cell responses. Lipopolysaccharide (LPS), muramyl-dipeptide (MDP), T-cell receptor and costimulation (αCD3/αCD28 coated beads) and IL-10 signalling blockade were used alone or in combination based on the concept that innate pathogen recognition receptor responses in particular, NOD2^14^and TLR4,^20^ T-cell responses and IL-10 signalling defects are implicated by multiple genetic IBD susceptibility loci and Mendelian forms of IBD.^21^ We performed microarray gene expression analysis of all conditions at 16 hours following stimulation in the presence or absence of IL-10 receptor (IL-10R) blocking antibodies. We identified stimulus-specific and shared gene expression signatures (online supplemental figure 1B, C and online supplemental table 2). LPS, L18MDP and αCD3/αCD28 coated beads induced 319, 187 and 312 genes, respectively, out of which 109, 19 and 175 were condition-specific genes (Benjamini-Hochberg (BH)-adjusted p<0.05, fc≥1.5; online supplemental figure 1B). Changes in *IL23A* expression were moderate under these conditions (online supplemental figure 1C). We next investigated the role of IL-10 signalling during LPS, L18MDP and αCD3/αCD28 stimulation. In total, 36 genes were upregulated and 29 genes were downregulated by IL-10 blockade, with most of the changes being found during LPS stimulation (fc>1.5, BH-adjusted p<0.05; online supplemental figure 1D, E and online supplemental table 3). *IL23A* expression was significantly upregulated under conditions of LPS or L18MDP stimulation and IL-10 signalling blockade (online supplemental figure 1C, D).

10.1136/gutjnl-2020-321731.supp2 Supplementary data

10.1136/gutjnl-2020-321731.supp3 Supplementary data

10.1136/gutjnl-2020-321731.supp4 Supplementary data

10.1136/gutjnl-2020-321731.supp5 Supplementary data

**Figure 1:**
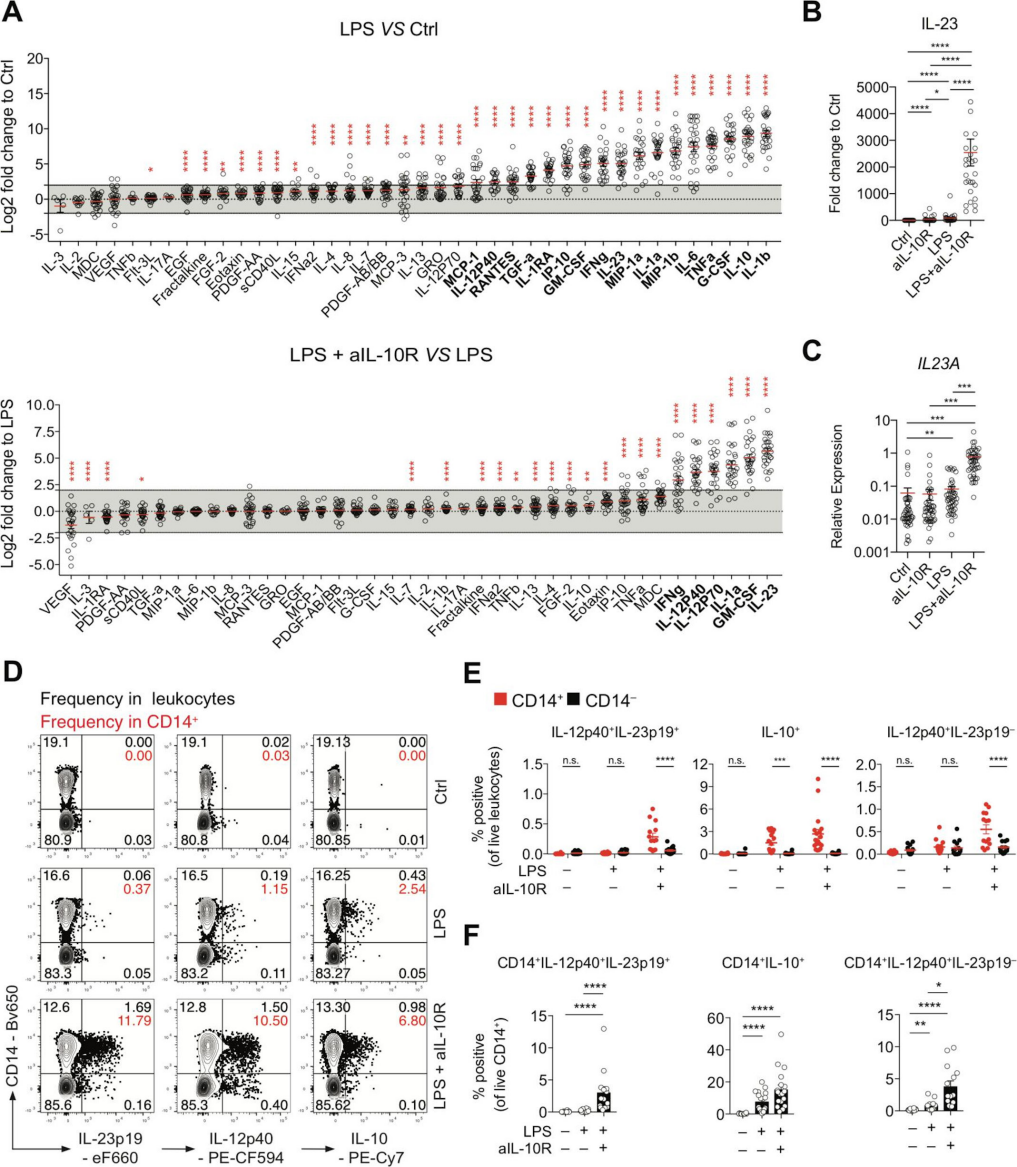
IL-10 regulates *IL23A* transcription and IL-23 protein secretion in a subset of monocytes from patients with IBD. (A) Analysis of PBMC culture supernatants collected after 16 hours of stimulation with LPS±IL-10R blocking antibodies expressed as log2 fold change to unstimulated PBMC (Ctrl) or LPS-stimulated PBMC (n=28; mean±SEM; Wilcoxon test, BH-adjusted p values). (B) IL-23 protein concentrations in culture supernatants expressed as fold change to unstimulated PBMC (n=28; mean±SEM; Friedman test with FDR-adjusted p values). (C) RT-qPCR analysis of relative *IL23A* expression in PBMC following 16 hours of stimulation (n=45; mean±SEM; Kruskal-Wallis test, BH-adjusted p values). (D) Contour plot presentation of IL-23p19-producing, IL-12p40-producing and IL-10-producing live leukocytes and CD14 surface expression measured at 16 hours poststimulation in PBMC. (E) Frequencies of IL-12p40^+^IL-23p19^+^, IL-10^+^ and IL-12p40^+^IL-23p19^–^ CD14^+^ and CD14^–^ cells in total live leukocytes (n=18, mean±SEM, Mann-Whitney test). (F) Frequencies of IL-12p40^+^IL-23p19^+^, IL-10^+^ and IL-12p40^+^IL-23p19^–^ of CD14^+^ cells (n=18, mean±SEM, Mann-Whitney test). BH, Benjamini-Hochberg; Ctrl, control; EGF, epidermal growth factor; FDR, false discovery rate; G-CSF, granulocyte-colony stimulating factor; IL, interleukin; LPS, lipopolysaccharide; MDC, macrophage-derived chemokine (CCL22); n.s., not significant; PBMC, peripheral blood mononuclear cells; RANTES, regulated upon activation - normal T cell expressed and presumably secreted (CCL5); RT-qPCR, real-time quantitative PCR; TNF, tumour necrosis factor. *pvalue 0.05, **pvalue<0.01, ***pvalue<0.001, ****pvalue<0.0001.

To investigate the regulation of secreted proteins by IL-10, we analysed cell culture supernatants after 16 hours of stimulation (figure 1A). LPS stimulation significantly upregulated protein secretion in 17 among the 40 proteins tested (fc≥4-fold, BH-adjusted p<0.05). The addition of IL-10 blockade significantly upregulated six of the LPS-induced factors (IL-23p19, GM-CSF, IL-1α, IL-12p70, IL-12p40 and interferon (IFN)-γ) (BH-adjusted p<0.05, fc≥4 fold) (figure 1A). Compared with control, LPS stimulation resulted in a mean 85.23-fold induction (mean concentration control: 3.30 pg/mL, mean concentration LPS: 135.98 pg/mL) of IL-23 (BH-adjusted p<0.05), while combined LPS and anti-IL-10R treatment induced a mean 2544.95-fold increased (mean concentration LPS and anti-IL-10R: 4737.57 pg/mL) IL-23 secretion (BH-adjusted p<0.05) (figure 1B). IL-23 mRNA and protein expression in PBMC were not correlated with disease subset or disease activity (online supplemental figure 2A‒D). Similarly, in previous studies, IL-23 did not emerge as a biomarker of disease phenotype (CD*/*UC/healthy controls) or disease activity in treatment-naïve or treated patients with IBD.^17 22 23^

10.1136/gutjnl-2020-321731.supp6 Supplementary data

We next sought to investigate IL-10 and IL-23 production at the single-cell level and analysed monocytes cytokine production by flow cytometry. At 16 hours poststimulation, monocytes produced IL-10 but low IL-12p40 and IL-23p19. The synthesis of all three proteins was significantly increased when cells were stimulated with LPS and anti-IL-10R. IL-23^+^ (IL-12p40^+^IL-23p19^+^) and IL-12^+^ (IL-12p40^+^IL-23p19^–^) cell frequencies and mean fold change were significantly greater in CD14^+^ relative to CD14^–^ leucocytes after IL-10 receptor blockade (IL-23: CD14^–^: fc=2.7, CD14^+^: fc=28.5; IL-12: CD14^–^: fc=1.1, CD14^+^: fc=3.5) (figure 1D‒F). These results were confirmed in PBMC obtained from healthy donors (HDs) (IL-23: CD14^–^: fc=7.3, CD14^+^: fc=13.3; IL-12: CD14^–^: fc=1.9, CD14^+^: fc=6.3) (online supplemental figure 3A‒C). Together, these results demonstrate that IL-10 signalling regulates IL-23 (IL-12p40^+^IL-23p19^+^) production in a subset of CD14^+^ monocytes (IBD mean=3.03%, 95% CI of the mean: lower=1.11, upper=4.96; HD mean=5.32%, 95% CI of the mean: lower=3.51, upper=7.14).

10.1136/gutjnl-2020-321731.supp7 Supplementary data

### Single-cell sequencing identifies inflammatory IL-10-regulated monocyte phenotypes

**Figure 2:**
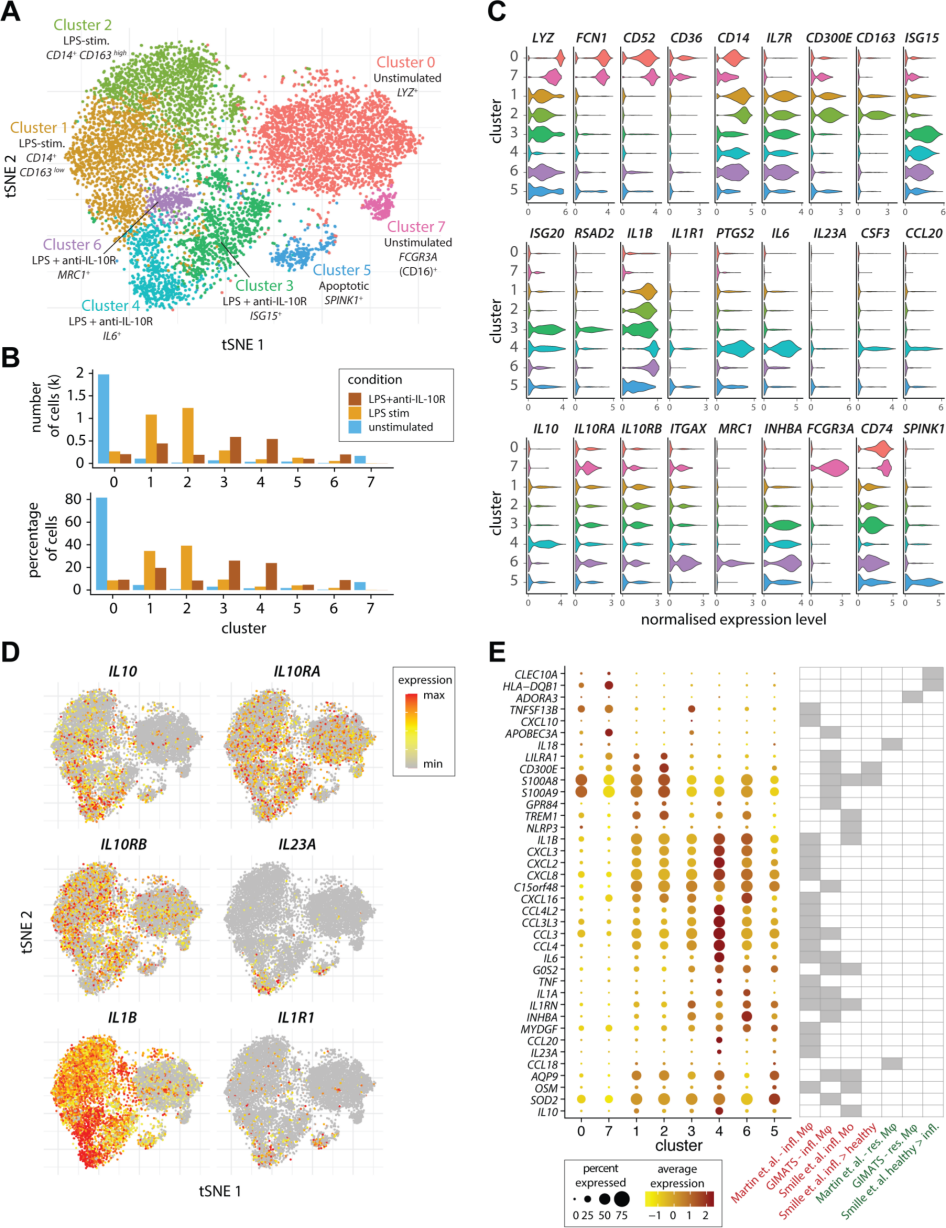
Single-cell RNA sequencing identifies subsets of inflammatory monocytes. (A) The tSNE plot shows the subpopulations of unstimulated, LPS-stimulated and combined LPS and anti-IL-10R-stimulated monocytes that were identified by a graph-based clustering approach following cross-condition alignment with Harmony (for details please see the online supplemetary materials and supplementary references^61^) (B) Bar plots show the number and frequency of cells in each of the identified clusters among three stimulation conditions in each of the identified clusters. (C) The violin plots show expression (x-axis) of genes characteristic of the identified monocyte clusters (y-axis). (D) Expression of *IL10*, *IL10RA*, *IL10RB*, *IL23A*, *IL1B and IL1R1* across the single monocytes according to (A). (E) The dot plot shows the expression of genes associated with Mo and Mφ in single-cell studies of CD (Martin *et al*)^9^ and UC (Smille *et al*)^25^ in the identified clusters of single cells (A). The genes shown were previously identified as characteristic of inflammatory macrophages in CD (‘Martin *et al* infl. Mφ’) or in anti-TNF-resistant CD (‘GIMATS–infl. Mφ’),^9^ as characteristic of inflammatory monocytes (‘Smille *et al* infl. Mo’) or higher in monocytes from inflamed versus healthy tissue (‘Smille *et al* infl.>healthy’) in UC,^25^ as characteristic of resident intestinal Mφ in the context of CD (‘Martin *et al*.–res. Mφ’ and ‘GIMATS–res. Mφ’),^9^ or more highly expressed in healthy than diseased tissue in the context of UC (‘Smille *et al* healthy>infl.’).^25^ For further details, please see the Methods section. CD, Crohn’s disease; GIMATS, IgG plasma cells - inflammatory mononuclear phagocytes - activated T cells - stromal cells; IL, interleukin; LPS, lipopolysaccharide; Mφ, macrophages; Mo, monocyte; TNF, tumour necrosis factor; tSNE, t-distributed stochastic neighbour embedding.

FACS analysis suggested the presence of unknown functional heterogeneity within the population of stimulated CD14^+^ monocytes (see *e.g*. figure 1D). To characterise the LPS-induced and IL-10-regulated transcriptional profile of monocytes subsets, and to differentiate population-wide transcriptional changes from subset-specific responses we performed single-cell RNA sequencing (scRNA-Seq) of unstimulated, LPS-stimulated and LPS and anti-IL-10R-treated CD14^+^ MACS-sorted monocytes from HDs (figure 2A). Flow cytometry analysis of the expression of CD14, CCR2 and CD16 by these cells prior to scRNA-Seq confirmed the presence of ‘classical’, ‘intermediate’ and ‘non-classical’ monocytes at the expected frequencies in each of the samples (online supplemental figure 4A–C). Among the eight clusters of monocytes that were detected, two clusters emerged after LPS stimulation and three additional clusters largely comprised of LPS and anti-IL-10R stimulated cells (figure 2A, B).

10.1136/gutjnl-2020-321731.supp8 Supplementary data

The eight clusters showed discrete gene expression (figure 2C and online supplemental figure 5) and were enriched for biological processes suggestive of different functional specialisations (online supplemental figure 6A). The majority of unstimulated monocytes were lysozyme*^+^* (*LYZ*), *CD52^+^* (*CAMPATH1*) and *FCN1* ^+^ expressing *CD14* ^high^ monocytes (cluster 0—unstimulated) typical of classical monocytes, while a minority of unstimulated monocytes displayed a *CD52* ^+^, *FCN1* ^+^, *CD16* ^+^ (*FCGR3A*) *CD14* ^low^ phenotype (cluster 7—unstimulated) characteristic of intermediate or non-classical monocytes in keeping with the flow cytometry data (online supplemental figure 4).^24^ Two clusters of cells that emerged following LPS stimulation were composed of *CD14* ^+^ *IL1B^+^*cells. While these two clusters of cells had very similar profiles (online supplemental figure 6B), the first showed lower expression of *CD163* and *CD300E* and was enriched for genes associated with ‘T-cell tolerance induction’ (cluster 1—LPS-stimulated), while the second displayed higher *CD163* and *CD300E* expression and an enrichment for ‘monocyte activation’ genes (cluster 2—LPS-stimulated). The three additional monocyte phenotypes that appeared on combined LPS stimulation and IL-10R blockade were demarcated by (1) high expression of type I IFN-responsive genes (eg, *IFIs*, *IFITs*, *ISGs*, *OASs* and *IRFs*) and genes linked to antigen processing and presentation (*e.g. B2M*, *CCR7*, *HLA* and *CD74*) (cluster 3—LPS and anti-IL-10R, called IFN-induced monocytes); (2) high expression of *ITGAX* and enrichment for genes associated with ‘superoxide generation’ and ‘positive regulation of neutrophil activation’ (cluster 6—LPS and anti-IL-10R, called microbicidal monocytes) and (3) expression of proinflammatory genes including (*IL1B*, *IL6*, *IL23A*, *CCL20* and *PTGS2*) and enrichment of genes associated with ‘Th17 cell lineage commitment’, ‘positive regulation of acute inflammatory response’ and ‘IFN-γ production’ (cluster 4—LPS and anti-IL-10R, called IL-23^+^ inflammatory monocytes). These three phenotypes in LPS-stimulated anti-IL-10R-treated monocytes were replicated in a second donor (online supplemental figure 6C).

10.1136/gutjnl-2020-321731.supp9 Supplementary data

10.1136/gutjnl-2020-321731.supp10 Supplementary data

More detailed examination of the expression of key cytokines and their receptors revealed that while *IL23A* and *IL1R1* were detected in a cluster-specific manner under the ‘hyperinflammatory’ LPS and anti-IL-10R condition, *IL10*, *IL10RA* and *IL10RB*, as well as *IL1A/IL1B* mRNA showed a broader, cross-condition expression (figure 2D). Finally, we sought to compare the monocyte phenotypes that we identified following ex vivo stimulation with those reported for colonic mononuclear phagocytes in human IBD. We show that many of the genes associated with inflammatory mononuclear phagocyte phenotypes in CD^9^ (‘inflammatory macrophages’) and UC^25^ (‘inflammatory monocytes’) were upregulated in clusters associated with LPS or LPS+anti-IL-10R stimulation (figure 2E). Clusters mainly composed of cells from the LPS+anti-IL-10R stimulation condition showed the highest induction of the cassettes of disease-associated cytokines and chemokines, with the *IL23A* ^+^ cluster 4 being marked by a particularly high expression of *IL6*, *IL1A*, *IL1B*, *TNF* and *OSM*, as well as elevated levels of *IL10* (figure 2D).

### IL-10-producing monocytes control IL-23-producing monocytes through paracrine signalling

**Figure 3:**
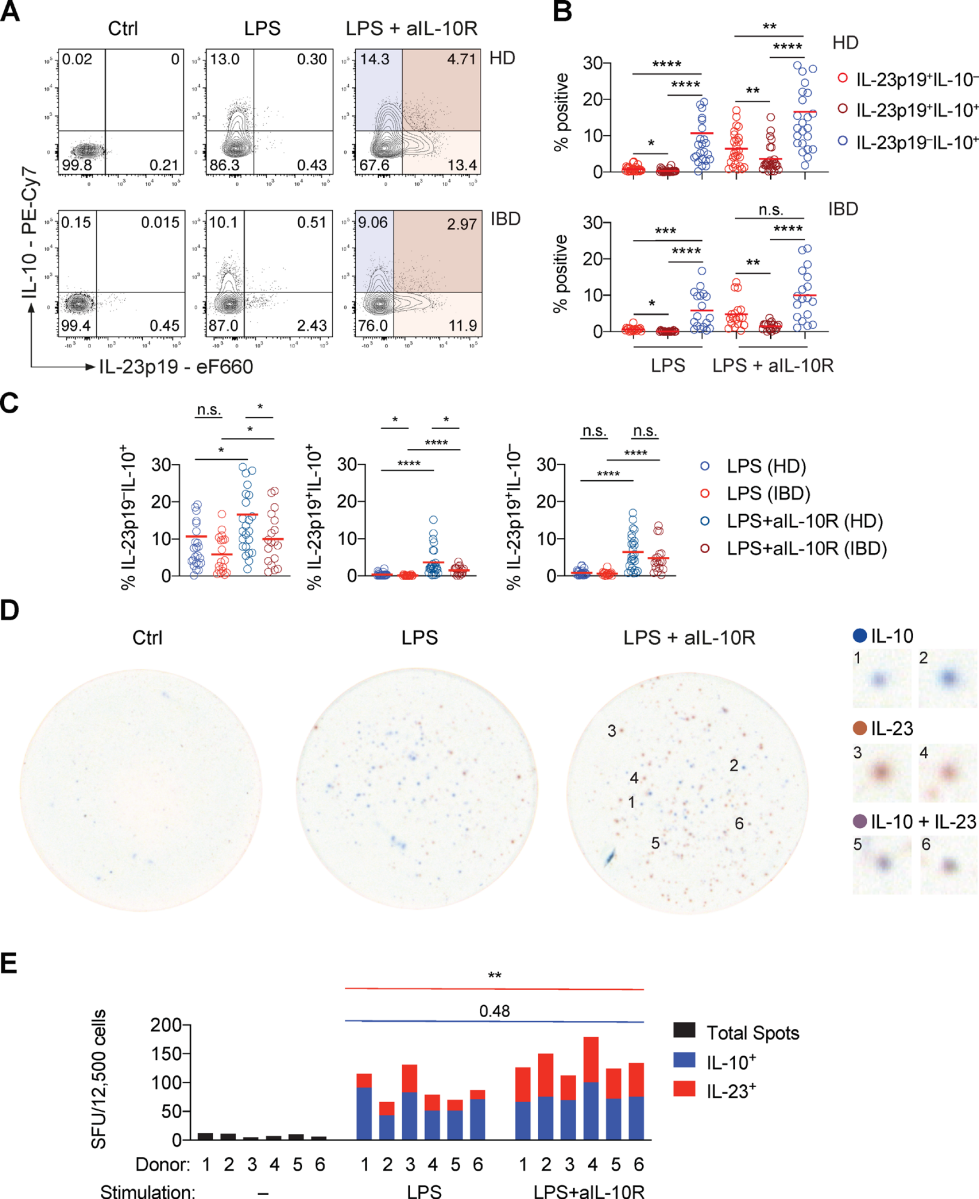
The monocyte response to PRR stimulation is heterogeneous, revealing a paracrine mechanism of IL-10-depedent regulation of IL-23 production. (A) Representative contour plot presentation showing IL-23p19^+^ and IL-10^+^ frequencies in monocytes derived from a healthy donor and a patient with IBD at 16 hours poststimulation. (B) IL-23p19^+^IL-10^–^, IL-23p19^+^IL-10^+^ and IL-23p19^–^IL-10^+^ monocyte frequencies at 16 hours poststimulation (HD: n=26, IBD: n=18; mean±SEM; Friedman test with FDR-adjusted p values). (C) Monocyte cytokine frequencies according to (A) and (B), comparing patient subgroups and stimulation conditions (HD: n=26, IBD: n=18; mean±SEM, Mann-Whitney test). (D) Representative dual-colour ELISpot images showing non-stimulated, LPS-stimulated and combined LPS and anti-IL-10R-stimulated monocyte IL-10 secretion (blue), IL-12p40^+^IL-23p19^+^ secretion (red) and combined production of both cytokines (violet/brown). Numbered magnifications of distinct spots detected in the LPS+anti-IL-10R-stimulated condition are shown on the right. (E) Dual-colour ELISpot counts from three independent experiments (n=6, Mann-Whitney test). Ctrl, control; FDR, false discovery rate; IL, interleukin; n.s., not significant; LPS, lipopolysaccharide; SFU, spot forming units. *pvalue<0.05, **pvalue<0.01, ***pvalue<0.001, ****pvalue<0.0001.

In PBMC, we observed that *IL23A* and *IL10* mRNA expression were strongly correlated (Spearmans’ r=0.84, p<0.0001) when stimulated with LPS and IL-10R blockade (online supplemental figure 7A). These data indicate that *IL10* mRNA expression itself is tightly regulated by IL-10 signalling as part of a negative feedback mechanism. We assumed that *IL23A* and *IL10* would be produced by the same cells. However, inspection of the single-cell data suggested that this might not be the case. Intracellular flow cytometry demonstrated that IL-10 expression in monocytes at the population level was earlier then the upregulation of IL-23 (online supplemental figure 7B) and that IL-10 and IL-23 largely originate from distinct cells in both monocytes isolated from HD and patients with IBD (figure 3A, B). While all subsets of IL-23p19 and/or IL-10-producing monocytes were deregulated on IL-10R blockade, monocytes isolated from patients with IBD showed decreased frequencies of IL-10^+^ single producing monocytes and IL-23p19^+^IL-10^+^ coproducing monocytes but not IL-23p19 single producing monocytes when compared with monocytes isolated from healthy donors (figure 3C). The presence of diverse cytokine profiles in monocytes indicated that IL-10 may regulate IL-23 production through a paracrine mechanism in functionally distinct cells. Alternatively, monocytes might express *IL10* and *IL23A* mRNA sequentially (ie, with early IL-10 producer becoming IL-23 producers). Otherwise, the pattern of segregated IL-10 and IL-23 protein expression might result from oscillatory expression of *IL10* and *IL23A* mRNA. To distinguish between these possibilities, we performed a dual-colour ELISpot assay using MACS-purified monocytes. This approach captured cytokine production in the individual cells over the full duration of their culture in vitro. In line with a paracrine model where IL-10-producing monocytes regulate IL-23 production in others, we found that IL-10-producing monocytes were distinct from IL-23-producing monocytes (figure 3D, E, and online supplemental figure 7C). These results suggest that the IL-10-producing monocytes regulated the IL-23-producing cells through a paracrine mechanism and that the observed monocyte heterogeneity was not the result of sequential or oscillatory cytokine secretion. The absence of aggregates of either IL-10-secreting or IL-23-secreting monocytes additionally demonstrated that the development of IL-23-producing monocytes did not result from local cell segregation in tissue culture.

10.1136/gutjnl-2020-321731.supp11 Supplementary data

### Deconvolution of intestinal IL-23 gene expression in intestinal tissue

**Figure 4:**
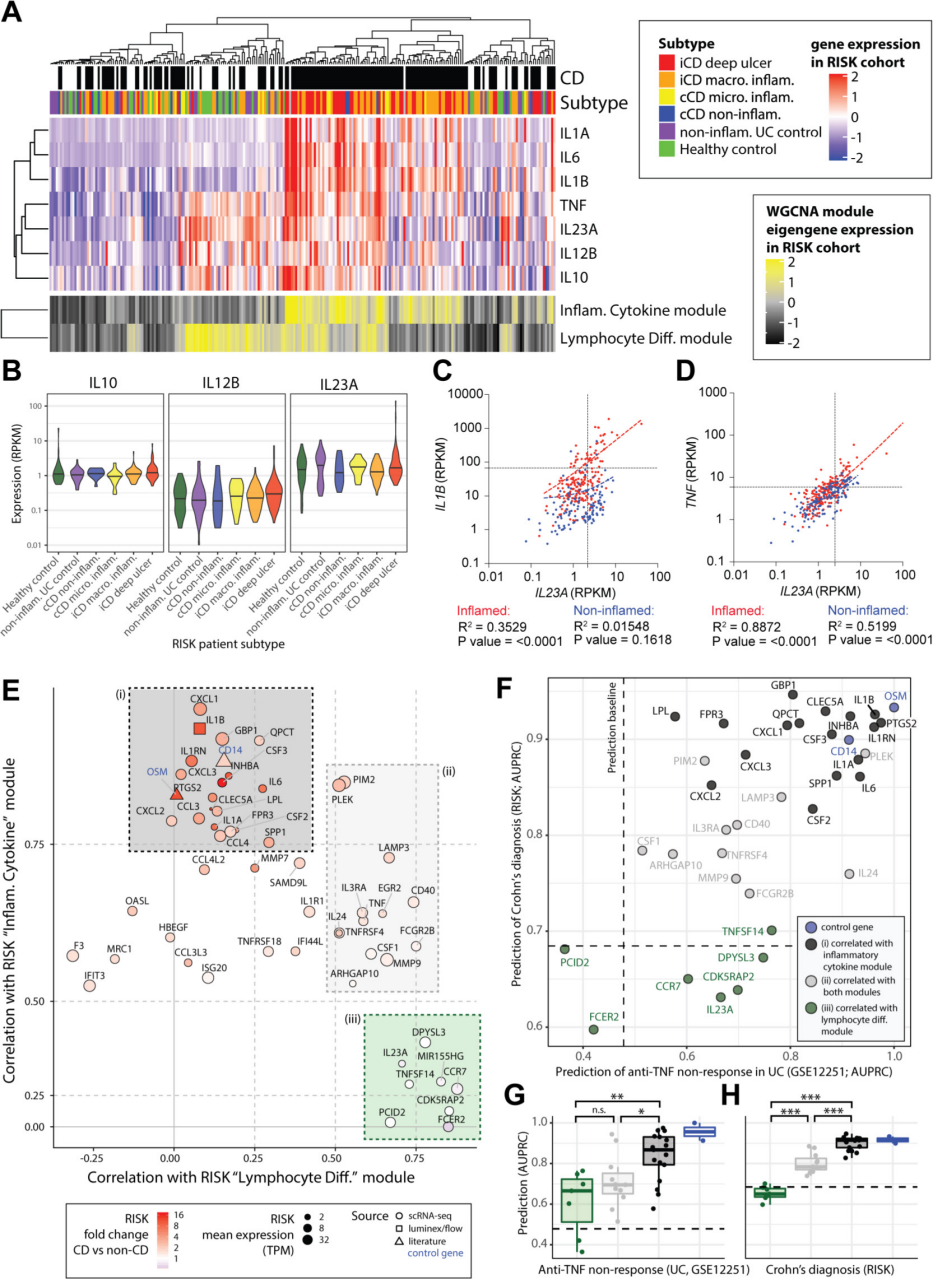
An IL-10-regulated inflammatory monocyte gene signature informs IL-23 and IL-1 targeting therapeutic approaches in IBD. (A) Patients of the RISK cohort (diagnosis and subtype as shown in top panels) were clustered according to the expression of 22 modules of coexpressed genes (see online supplemental figure 7A). The upper heatmap shows expression of key cytokines across the cohort. The lower panel shows expression of the eigengenes of two of the identified modules of coexpressed genes. (B) The expression of *IL10*, *IL12B* and *IL23A* within the different patient strata of the RISK cohort. (C, D) Correlation of *IL23A* with *IL1B* (C) and *IL23A* with *TNF* (D) in the inflamed (red) and non-inflamed (blue) CD patient biopsies of the RISK cohort. (E) Correlation of genes specific to LPS and anti-IL-10R-stimulated monocytes with the ‘inflammatory cytokine’ and ‘lymphocyte differentiation’ gene modules in the RISK cohort data identifies three subsets (shaded (i) black, (II) grey and (III) green boxes). (F) Assessment of the ability of the identified monocyte genes to predict anti-TNF non-response (x-axis, GSE12251) and diagnosis of Crohn’s (y-axis, RISK cohort). The dashed lines indicate random classifier performance. (G, H) Comparison of the ability of the identified subsets of monocyte genes to predict TNF non-response (GSE12251) and diagnosis of CD in the RISK cohort (Wilcoxon tests, colours as shown in panels E, F). AUPRC, area under precision recall curve; CD, Crohn’s disease; IL, interleukin; LPS, lipopolysaccharide; n.s., not significant; RISK, Risk Stratification and Identification of Immunogenetic and Microbial Markers of Rapid Disease Progression in Children with Crohn’s Disease study; TNF, tumour necrosis factor; WGCNA, weighted gene correlation network analysis. *pvalue<0.05, **pvalue<0.01, ***pvalue<0.001, ****pvalue<0.0001.

We next sought to understand the context of IL-23 expression during intestinal inflammation. We compared expression of IL-23 in ileal biopsies from 219 patients with CD and non-inflamed controls in samples from the Risk Stratification and Identification of Immunogenetic and Microbial Markers of Rapid Disease Progression in Children with Crohn’s Disease(RISK) study 26 (controls without ileal inflammation include 42 healthy donors and 61 patients with UC) (figure 4A). As expected, *IL23A* and *IL12B* were expressed in the CD patient samples, along with *IL1A*, *IL1B* and *IL6*. However, we also found *IL23A* and *IL12B* expression in non-inflamed control biopsies (figure 4A, B) where *IL23A* expression was associated with *TNF* rather than *IL1B* expression (figure 4C, D). We therefore performed weighted gene correlation network analysis^27^ to investigate the possible sources and roles of *IL23A* in the inflamed and non-inflamed intestines (online supplemental figure 8A). This analysis identified 22 modules of coexpressed genes that were named according to their gene members and enrichments for biological pathways and sets of cell-type marker genes (online supplemental table 4). Clustering of the modules by their expression patterns in the RISK cohort patients identified six major groups that could be broadly distinguished by their correlations with diagnosis of CD, *IL23A* expression, epithelial gene expression, mitochondrial activity and cell growth genes (online supplemental figure 8A, B).

10.1136/gutjnl-2020-321731.supp12 Supplementary data

10.1136/gutjnl-2020-321731.supp13 Supplementary data

Most prominently, we found an ‘inflammatory cytokine’ module that was significantly correlated with both CD (r=0.55) and *IL23A* expression (r=0.35) (online supplemental figure 8B). This module contained key myeloid and stromal markers genes (*CD14* and *PDPN*), proinflammatory cytokines (including *OSM*, *IL1B* and *IL6)* and fibroblast activation protein (online supplemental figure 8B) in keeping with the emerging concept of a pathogenic myeloid-stromal cell circuit in IBD.^18 24^ Intriguingly, however, we found that *IL23A* expression was most strongly correlated with two modules of ‘immune cell differentiation’ (r=0.7) and ‘lymphocyte differentiation’ (r=0.71) genes (online supplemental figure 8A). Of these, the immune cell differentiation module was weakly correlated with CD (r=0.2), associated with expression of myeloid (*CD14)* and lymphoid (*CD79A*, *CD4*) cell markers, and enriched for ‘myeloid DC differentiation’ (online supplemental figures 8B and 9A). The lymphocyte differentiation module showed an expression signature suggestive of lymphoid follicles, including individual markers of B-cell and T-cell identity (*CD79A* and *CD4*) and differentiation (*BACH2)* 25 26 (online supplemental figure 8B), as well as enrichments for gene ontology categories related to lymphoid cell differentiation, proliferation and selection (online supplemental figure 9A). While the lymphocyte differentiation module was not correlated with CD, it was unique in showing significant enrichments for two sets of IBD Genome-wide association study (GWAS)-associated genes (OR>3), suggesting that this set of genes is relevant for the pathogenesis of IBD (online supplemental figure 9B). We also noted that both of the *IL23A-*associated immune cell differentiation and lymphocyte differentiation modules were significantly enriched for genes belonging to the KEGG ‘Th17 cell differentiation’ pathway (online supplemental figure 9A).

10.1136/gutjnl-2020-321731.supp14 Supplementary data

Overall, the network analysis suggested that *IL-23* expression was primarily associated with lymphoid cell differentiation in both health and disease. We therefore sought to understand which of the IL-10-regulated monocyte genes were specific to disease-associated inflammation. We correlated the expression of a curated set of IL-10-regulated monocyte genes (35 genes specific to combined LPS and anti-IL-10R stimulation scRNA-Seq; figure 4E, F; online supplemental methods section, ‘Identification and characterisation of an IL-10-responsive monocyte gene signature’) with the eigengenes for the CD-associated inflammatory cytokine and the non-inflammatory lymphocyte differentiation modules in the RISK cohort data. This analysis identified three subsets of IL-10-regulated monocyte genes (figure 4E). These comprised (1) genes correlated only with the CD associated inflammatory cytokine module (black box, figure 4E); (2) a set of genes correlated with both the inflammatory cytokine and lymphocyte differentiation modules (grey box, figure 4E); and (3) genes correlated with only the non-inflammatory lymphocyte differentiation module (green box, figure 4E). The IL-10-regulated genes that correlated with the inflammatory cytokine but not the lymphocyte differentiation eigengene (black box, figure 4E) showed a significantly superior ability to predict anti-TNF response after 4–6 weeks of treatment in an independent cohort of patients with UC (UC-cohort GSE12251)^28^ and diagnosis of CD in the RISK cohort^26^ (figure 4F‒H, online supplemental table 5). By contrast, IL-10-regulated monocyte genes that correlated with lymphocyte differentiation, but not inflammatory cytokines, had poorer predictive ability (figure 4F‒H). To validate our findings, we analysed intestinal transcriptome data of an additional adult cohort of patients with CD and UC (GSE16879).^28^ Similar to our initial results, inflammatory monocytes gene expression predicted disease activity and anti-TNF non-response in adult patients with CD ileal biopsy transcriptomes. In colonic biopsies, these genes were not predictive of disease activity but distinguished patients with CD or UC anti-TNF non-response (online supplemental figure 10A, B).

10.1136/gutjnl-2020-321731.supp15 Supplementary data

10.1136/gutjnl-2020-321731.supp16 Supplementary data

These data therefore identify a subset of IL-10-regulated monocyte-derived genes, including *CXCL1/2*, *IL1A*, *IL1B*, *INHA*, *IL6*, *CCL3/4*, *PTGS2*, *CSF2/3* and *GBP1*, which show a specific association for disease-associated intestinal inflammation in the RISK and GSE16879 CD cohorts. By contrast, cytokines such as IL-23 and TNF were not specific for disease suggestive of context-dependent roles in homeostasis and inflammation.

### Monocyte exposure to whole bacteria causes aquired IL-10 resistance and IL-23 secretion

**Figure 5:**
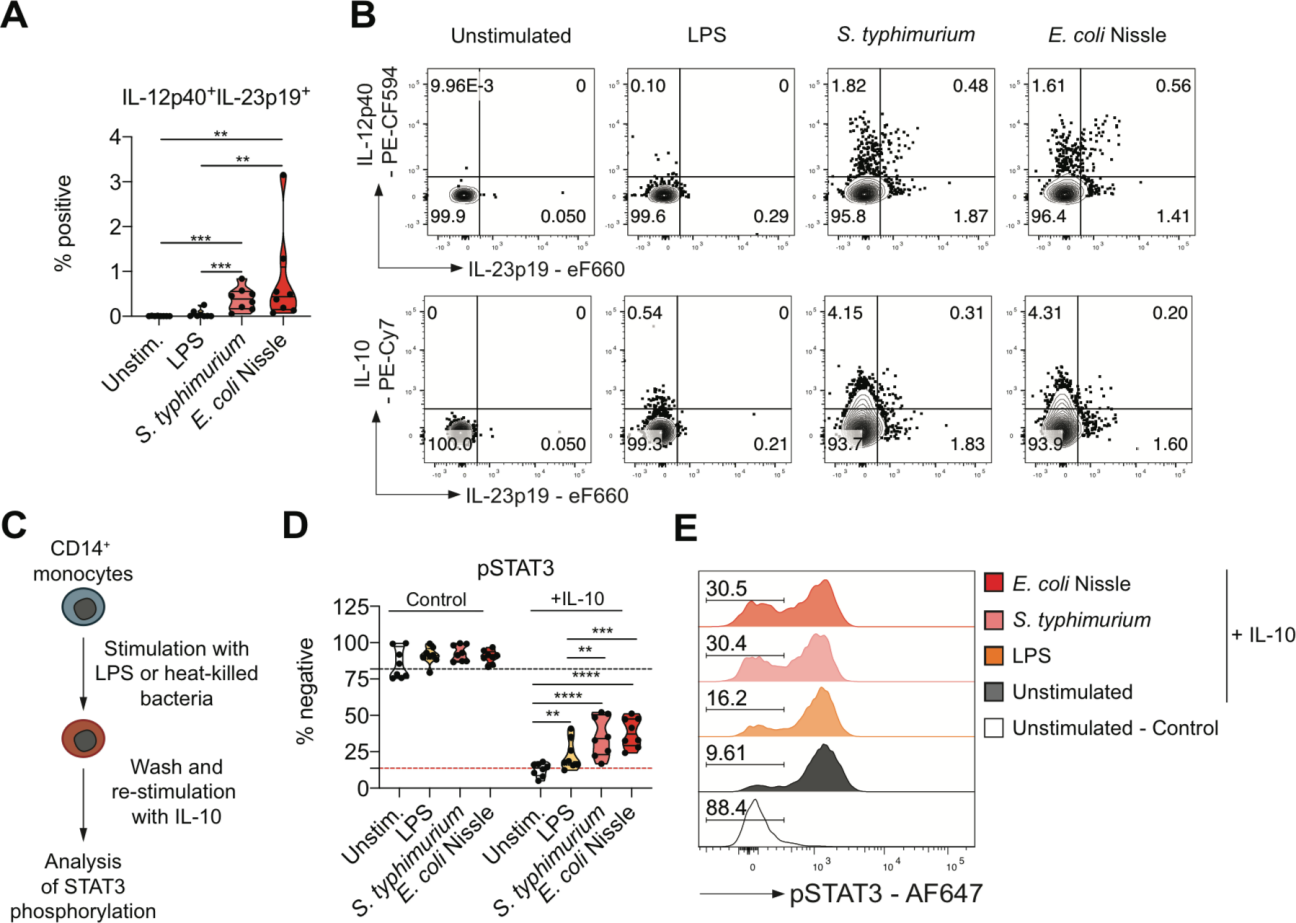
Monocyte stimulation with whole bacteria causes IL-10 resistance and IL-23 secretion. (A) Frequencies of IL-12p40^+^IL-23p19^+^ in live CD14^+^ at 16 hours following stimulation (n=8; mean±SEM, Mann-Whitney test). (B) Dot plots showing IL-12p40, IL-23p19 and IL-10 in live CD14+ monocytes from a healthy donor. (C) Scheme for the assessment of monocyte IL-10 responsiveness. (D) Frequencies of phospho-STAT3^–^ live monocytes without cytokine stimulation (control) and following 15 min IL-10 (50 ng/µL) stimulation (n=8; mean±SEM, Mann-Whitney test). (E) Representative histograms showing phosphorylation of STAT3 in non-treated (control) or IL-10-treated (+IL-10) live monocytes. Percentages of IL-10 stimulation-resistant monocytes are indicated. IL, interleukin; LPS, lipopolysaccharide. *pvalue<0.05, **pvalue<0.01, ***pvalue<0.001, ****pvalue<0.0001.

The analysis of gene expression data in the RISK cohort suggests IL-23 expression despite the presence of IL-10 in a subset of patients with macroscopic inflammation and deep ulcerating IBD in the absence of Mendelian IL-10 or IL-10R loss of function DNA variants, that is, functional IL-10 resistance. In light of the epithelial barrier defect in ulcerating disease that predisposes to bacterial translocation, we tested whether exposure of monocytes to bacteria can induce functional IL-10 resistance. We treated monocytes for 16 hours with heat killed *Salmonella typhimurium* or *Escherichia coli* strain Nissle. Interestingly, monocytes produced IL-23 and IL-10 on stimulation (figure 5A, B) reminiscent of LPS stimulation in presence of IL-10R blockade. The cellular dichotomy of IL-23 and IL-10 production was maintained under these conditions (figure 5B). To assess IL-10 responsiveness in previously stimulated monocytes, we extensively washed cell cultures, subsequently exposed those to recombinant human IL-10 and evaluated phosphorylation of signal transducer and activator of transcription (STAT)3 (figure 5C). Indeed, bacterial stimulation was associated with an increased proportion of IL-10 non-responsive cells, demonstrating that monocyte exposure to whole bacteria induced a state of acquired IL-10 resistance (figure 5D, E). Importantly, the reduced phosphorylation of STAT3 in monocytes that were exposed to whole heat killed bacteria was not due to increased cell death under these culture conditions (online supplemental figure 11A, B).

10.1136/gutjnl-2020-321731.supp17 Supplementary data

### IL-1α and IL-1β are essential for monocyte IL-23 production

**Figure 6:**
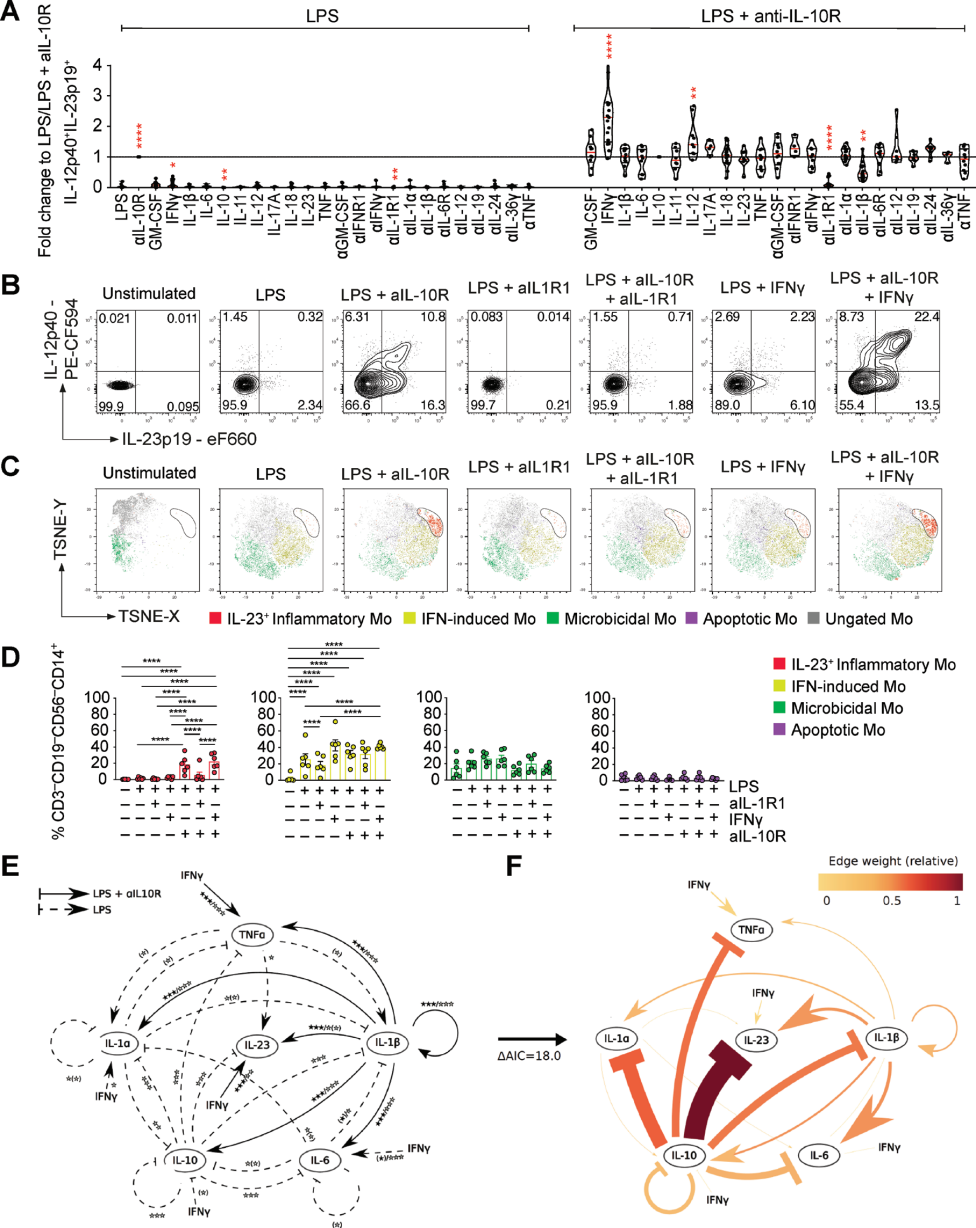
IL-1α and IL-1β signalling are essential for monocyte IL-23 production. PBMC from healthy donors (n>4) were stimulated for 16 hours with combinations of LPS and aIL-10R in the presence of indicated exogenous human recombinant cytokine (all 10 ng/mL) and/or cytokine/cytokine receptor blockade (all antibodies 10 µg/mL). (A) Frequencies of IL-12p40^+^IL-23p19^+^ live CD14^+^ monocytes (Wilcoxon test, 95% CI). (B) Representative dot plot showing intracellular IL-12p40 and IL-23p19 according to (A). (C) tSNE presentation of IL-23p19, CCL20, HLA-DR, IDO-1, CCL2, S100A8, RPS6 and SPINK-1 expression in live CD14^+^CD3^–^CD19^–^CD56^–^-gated monocytes. Analyses of three healthy donors are shown as overlay. (D) Frequencies of monocyte clusters across stimulations based on cluster-specifying protein expression (n=6; one-way analysis of variance after BH correction). (E) The extensive model represents the effects of the addition or blockade of cytokines in a PBMC culture in the presence of LPS or LPS and anti-IL-10R. Differences of cytokine addition or blockade in LPS-stimulated samples (dashed arrows), in LPS and anti-IL-10R-stimulated samples (solid arrows) and in LPS and anti-IL-10R-stimulated and anti- IL-1β treated conditions (dotted arrows). Nominal effects with p>0.05 after false discovery rate correction (BH) are shown in parentheses. (F) A reduced complexity model was established by focusing on informative cytokine interactions. Edge weights are defined as the relative contribution to model fit and are dependent on the network configuration considered. The edges of the 20-edge model have been coloured based on their weight. BH, Benjamini-Hochberg; IFN, interferon; IL, interleukin; LPS, lipopolysaccharide; PBMC, peripheral blood mononuclear cells; TNF, tumour necrosis factor.

The regulation of monocyte-derived IL-23 and IL-10 suggested coregulation via additional factors (likely cytokines). We therefore investigated the functional effects of 11 coregulated cytokines on monocyte IL-23 production (figure 6A, B and online supplemental figure 12A, B). Strikingly, when blocking IL-1R1, IL-23 production was near completely abolished (figure 6A, B and online supplemental figure 12A‒C). Blocking IL-1β or IL-1α alone had only partial or no effect on IL-23 production, indicating that either cytokine can compensate for the absence of the other in driving monocyte IL-23 production (figure 6A and online supplemental figure 12A). The effect of IL-1β on IL-23 expression was context specific, since addition of IL-1β in the presence of anti-IL-10R blockade alone did not induce IL-23 expression (online supplemental figure 12D). None of the other tested cytokines (GM-CSF, IL-6, IL-11, IL-17A, IL-18, IL-19, IL-23, IL-24, IL-36γ, TNF and type I IFN) demonstrated a significant impact on IL-23 production. Although not essential, addition of IFN-γ or IL-12 significantly increased monocyte IL-23 production (figure 6A, B and online supplemental figure 12A, B). To investigate the effects of IL-1R1 blockade on functional monocyte clusters, we stimulated PBMC from HDs with combinations of LPS and anti-IL-10R and analysed monocyte metaclusters-associated protein expression. In line with the single-cell mRNA sequencing experiments, the predicted clusters associated with LPS and combined LPS and anti-IL-10R stimulation (figure 6C). Blockade of IL-1R1 specifically inhibited the development of IL-23-producing monocytes, while other monocyte clusters remained largely unaffected (figure 6C, D). Importantly, monocyte IL-23 production induced by whole bacteria exposure was similarly dependent on IL-1R1 signalling (online supplemental figure 13A, B), suggesting that IL-23 is downstream of IL-1 signalling also in the context of bacteria-induced acquired IL-10 signalling defects.

10.1136/gutjnl-2020-321731.supp18 Supplementary data

10.1136/gutjnl-2020-321731.supp19 Supplementary data

We next used a mathematical model of ordinary differential equations as an objective analysis tool to investigate the relative direct and indirect impacts of the addition or blockade of various cytokines or blockade of cytokine receptors (figure 6E). The model tests whether cytokine production is modulated by other key cytokines that are produced by monocytes (TNF, IL-1α, IL-1β, IL-6, IL-10 and IL-23; false discovery rate (FDR)-corrected paired Wilcoxon test). Modelling all feasible network configurations led to the conclusion that most of the n=2^31-1^ configurations could be excluded a priori. We identified an optimal network describing the core network dynamics by ranking the models based on their fit to the data using the Akaike information criterion (figure 6F). This model supported IL-10 signalling as negative feedback mechanism and IL-1β as a positive regulator of IL-23 production, with IFN-γ acting as an amplifier. In addition, the model predicted the positive effect of IL-1β on IL-23 production to be independent of regulating IL-1α, IL-6, IL-10 or TNF and the negative regulation of IL-23 by IL-10 to be independent of the effect of IL-10 on the other cytokines present in the model (IL-1α, IL-1β, IL-6 and TNF). Interestingly, the model suggests that IFN-γ might amplify IL-1α through the suppression of IL-10, but amplify IL-23 independently of its inhibitory effect on IL-10 (the upregulatory edge from IFN-γ to IL-1α present in figure 6E was removed in the final model since this can be accounted for by a decrease in IL-10; figure 6F). Finally, negative regulation of IL-23 by IL-10 is predicted to be independent of the effect of IL-10 on the other modelled cytokines.

### IL-1 regulates IL-23 production in genetic IL-10 signalling defects

**Figure 7:**
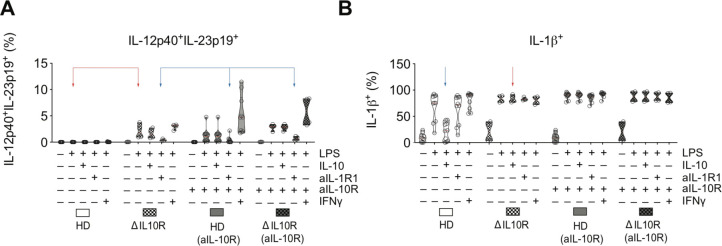
Monocyte IL-23 responses in patients with loss of function in IL-10R1 and IL-10R2. (A) Frequencies of IL-12p40^+^IL-23p19^+^ and IL-1β^+^ monocytes in PBMC from healthy donors and two patients with IL-10R variants (*IL10RA* (p.R117H) and *IL10RB* (p.Y167C)) following 16 hours of stimulation with combinations of LPS (200 ng/mL), IL-10 (10 ng/mL), anti-IL-10R (10 µg/mL), anti-IL-1R1(10 µg/mL) and IFN-γ (10 ng/mL). IFN, interferon; IL, interleukin; LPS, lipopolysaccharide; PBMC, peripheral blood mononuclear cells.

To confirm these predictions, we investigated the effect of IL-1R1 blockade on IL-23 expression in the context of genetic deficiency of IL-10 signalling. We stimulated PBMC obtained from patients with infantile-onset IBD due to an *IL10RA* or *IL10RB* gene defect. Interestingly, patient-derived monocytes produced IL-23 in response to LPS stimulation alone, indicating the intrinsic defect in IL-10R-dependent regulation, confirmed by the inability of exogenous IL-10 to suppress monocyte IL-1β production. Strikingly, IL-1R1 blockade inhibited monocyte IL-23 production in LPS-stimulated PBMC from IL-10R deficient patients (figure 7A, B). Together, these analyses confirmed IL-10 as the major negative regulator of IL-23 production, while IL-1 signalling (and in particular IL-1β) is essential for monocyte IL-23 synthesis.

## Discussion

We identify key regulatory circuits of IL-23 production by inflammatory monocytes. These include failure of paracrine IL-10-mediated control, as well as autocrine and paracrine signalling of IL-1α and IL-1β in response to inflammatory stimuli. Indeed, increased IL-23 production can be observed in monocytes from patients with infantile-onset Mendelian IL-10 signalling deficiency or experimental blockade of the IL-10 receptor. In addition, we newly show that monocytes that respond to whole bacteria express IL-23 as a consequence of acquired IL-10 non-responsiveness. Most importantly, we define a transcriptional signature of *IL23A* ^+^ inflammatory monocytes that indicates a state of acquired IL-10 non-responsiveness in a subgroup of patients with deep ulcers and compromised epithelial function where monocytes may directly respond to whole bacteria. This signature predicts both diagnosis of CD and resistance to anti-TNF treatment similar to previously described OSM expression.^18^ Although derived from analysis of ileal inflammation in CD, this signature was also able to predict anti-TNF failure from the transcriptional profiles of colonic samples in UC, suggesting a common role for hyperinflammatory monocytes in treatment-resistant disease in both IBD subtypes. Interestingly, loss of IL-10 responsiveness induces *IL23A* mRNA and IL-23 protein expression only in a small fraction of monocytes that express the IL-1 receptor *IL1R1*. This selective IL-23 expression contrasted with pervasive induction of IL-1α and IL-1β expression, as well as a broad induction of IL-10 expression in monocytes under the same hyperinflammatory condition.

Our data support context-specific roles of IL-23 in driving the differentiation of pathogenic Th17/Th1 cells in the presence of IL-1^29^ that are enriched in inflamed intestinal tissue from patients with IBD^30 31^ while supporting the development of non-pathogenic Th17 cells under homeostatic conditions such as those found in mucosa-associated lymphoid tissue (MALT) - in healthy individuals.

Together, these results suggest that subgroups of patients with active disease may benefit from anti-IL-23p19 therapy and that the targeting of upstream cytokines that regulate IL-23 expression in inflammation, such as IL-1 might provide a selective means to block pathogenic IL-23 expression. The critical element that differentiates homeostatic IL-23 and TNF expression from hyperinflammation is the coexpression of a multitude of IL-10-regulated factors, including IL-1α and IL-1β in patients with severe active disease and anti-TNF treatment non-responsiveness.

The strong predictive ability of an IL-10-sensitive inflammatory monocyte signature (*CD14*, *IL1A*, *IL1B, OSM*, *PTGS2*, *IL6*, *CCL2/3* and *CXCL1/2*) emphasises a surprising and underestimated extent of IL-10 non-responsiveness in IBD. In those patients, inflammation is present despite transcription of IL-10 (patients express a large amount of IL-10) and absence of pathogenic variants within the IL-10 receptor (exome sequencing did not reveal Mendelian forms of IL-10 receptor signalling defects in this cohort).^32^ The high expression of *PTGS2* in this subgroup of patients with IBD represents an additional indicator for deregulated IL-10 responses and intestinal antimicrobial immunity.^33^ Our experiments suggest that direct contact of monocytes with whole bacteria can induce IL-10 resistance that allows coexpression of IL-23 and IL-1 explaining the signature of IL-10 non-responsiveness in a subgroup of patients with intestinal ulceration. IgG receptor IIa signalling in myeloid cells in response to immunoglobulin coated microbes may additionally amplify inflammasome-dependent induction of IL-1 synthesis.^34^ An additional mechanism of reduced IL-10 responsiveness may be differential expression of IL-10R1.^35 36^ Our ELISpot experiments exclude differential spatial clustering of IL-10-secreting or IL-23-secreting monocytes, as well as oscillatory or sequential IL-10 and IL-23 transcription.

This suggests that the inflammatory microenvironment drives in parallel several monocyte effector programmes and monocyte to macrophage and DC differentiation processes, reminiscent of plasmacytoid DC stimulation with a single stimulus (R848 or CpG) that induces diverse transcriptional states and cellular functions.^37^ This is likely a biological mechanism to ensure functional heterogeneity under hyperinflammatory conditions, when diversity in the host defence towards a range of potential pathogens is essential.

Single-cell RNA transcriptomic approaches identified transcriptional signatures in circulating and tissue human monocytes and macrophages,^9 25 38–42^ demonstrating phenotypical and functional diversity in monocyte, macrophage and DC populations. We have focused on the cellular heterogeneity and inflammatory response of peripheral monocytes because those cells are recruited into the gut and differentiate into proinflammatory major histocompatibility complex (MHC)-II-high monocytes and macrophages that outnumber resident, yolk sac-derived tissue macrophages during inflammation.^43–45^ Such proinflammatory macrophages have been characterised following in vitro differentiation using LPS stimulation and express IL-12 and IL-23 in response to STAT1-dependent IFN-γ signalling.^46^

Recently *IL1Β^+^* ‘inflammatory monocyte’^25^ and *IL1B* ^+^ *IL23A* ^+^ ‘inflammatory macrophage’^9^ populations associated with inflammation and resistance to anti-TNF treatment have been described in single-cell studies of tissue from patients with UC^25^ and CD.^9^ We found that, following stimulation with LPS in the presence of anti-IL-10R blockade, a fraction of blood monocytes acquired a *IL1B* ^+^ *IL23A* ^+^ transcriptional signature that is very similar to those described for mononuclear phagocytes isolated from the inflamed intestinal biopsies of patients with CD and UC. This observation demonstrates the relevance of functional studies of peripheral monocytes for understanding the behaviour of these cells following their recruitment into the inflamed intestinal mucosa. Together with the previous findings, our observations suggest that inflammatory monocytes and CX3CR1^+^IL-1β^+^ macrophages^47 48^ are likely to be major sources of IL-23 in the context of inflammasome activation and IL-1 production in the inflamed intestine and support a critical role for IL-1 in intestinal anti-TNF-treatment refractory inflammation.

Importantly, several cell types including regulatory T cells, B cells, macrophages and DCs produce IL-10^49^ and may therefore contribute to the negative regulation of proinflammatory IL-23-producing monocytes in tissue in vivo. While we have unravelled the regulation of peripheral blood monocytes in IL-23 responses ex vivo and related those to processes of inflammation in intestinal tissue from patients with IBD, in the tissue context complex and niche-specific cellular communication networks are expected to further modulate the regulation of monocyte inflammatory effector functions and IL-23 expression.

In individuals without intestinal inflammation, our network analysis suggested that *IL23A* expression was likely derived from terminal ileal MALT because the lymphocyte differentiation to which it was assigned also showed strong correlations with orthologues of genes associated with murine small intestine lymphoid tissue (SILT), including *IL22RA2*, *ITGAX* 27 as well as with *VCAM1*, which is a marker of lymphoid-associated villi in humans (online supplemental figure 5B). In such MALT tissue, which is hyperplastic in paediatric patients,^50^ the source of *IL23A* is most likely DCs, tolerogenic CD103^+^DC^51^ or macrophages as is known to be the case in mouse SILT.^47^ This work demonstrates the utility of network-based approaches for deconvolving variance in gene expression that arises from the analysis of biopsy samples from tissues with complex microanatomical heterogeneity such as the small intestine.

Our mathematical model pinpoints IL-1 as a key positive regulator of IL-23 expression in monocytes. Studies in monogenic forms of IBD suggest that IL-1R1 blockade can resolve intestinal inflammation in patients with IL-10 receptor defects,^52^ mevalonate kinase defects and potentially gain-of-function NLRC4^53^ defects, which are all characterised by increased inflammasome activation and IL-1 secretion. It is therefore an attractive hypothesis that the hyperinflammatory IL1^+^PTGS^+^IL6^+^ signature might define an anti-IL-1R1 responsive group of patients with IBD. Interventional studies are required to identify differential effects between IL-23 and IL-1 blockade since both have complex effects on licensing for effector cytokine production and differentiation of Th1 and Th17 cells.^54^ Altogether, our findings may inform transcriptional diagnostic efforts to guide use of IL-23p19 targeting therapies and suggest a role for therapeutics capable of selectively blocking IL-23 in disease by targeting context-specific upstream factors such as IL-1.

10.1136/gutjnl-2020-321731.supp20 Supplementary data

## Data Availability

The data supporting the findings described in this study are available from the corresponding author upon request. Single-cell RNA-sequencing analysis of the IL-10 dependend response of human CD14+ monocytes to LPS have been deposited at NCBI’s Gene Expression Omnibus and are accessible under GEO Series accession number GSE130070. Microarray gene expression analysis of PBMC from patients with IBD stimulated by combinations of LPS, MDP, anti-CD3/anti-CD28 antibodies and anti-IL-10R blocking antibodies have been deposited at NCBI’s Gene Expression Omnibus and are accessible under GEO Series accession number GSE137680. Both data sets are available via a GEO SuperSeries that represents the publication as a whole (GSE138009). The links to the deposited data are link to the GEO Superseries (https://www.ncbi.nlm.nih.gov/geo/query/acc.cgi?acc=GSE138009), link to the microarray data (https://www.ncbi.nlm.nih.gov/geo/query/acc.cgi?acc=GSE137680), link to the scRNA-seq data (https://www.ncbi.nlm.nih.gov/geo/query/acc.cgi?acc=GSE130070).
