# Supplementary material for: Deconvolution of monocyte responses in inflammatory bowel disease reveals an IL-1 cytokine network that regulates IL-23 in genetic and acquired IL-10 resistance
Source: Gut. 2020 Oct 9;70(6):1023–36. doi: 10.1136/gutjnl-2020-321731 (PMC8108288; doi:10.1136/gutjnl-2020-321731)
Supplement: Supplementary data [file gutjnl-2020-321731supp001.pdf]

SUPPLEMENTARY FIGURE LEGENDS

Supplementary Figure 1: (**A**) Graphical illustration of ex vivo PBMC stimulations and experimental conditions. (**B**) Venn-diagram of differentially regulated genes in the three stimulation conditions (LPS, L18MDP, and αCD3/αCD28 coated beads). (**C**) Microarray analysis of patients with IBD (n=41; Supplementary Table 1) PBMC samples under diverse stimulation conditions. The heatmap shows transcripts found to be significantly differentially expressed (columns, fold change > 1.5, BH-adjusted pvalues<0.05) in the patient samples (rows) across diverse stimulation conditions (LPS, L18MDP and αCD3/αCD28) at 16 hours post stimulation. *IL23A* and *IL10* expression are shown on the right as log2 fold change expression to the unstimulated PBMC (Ctrl) median expression. (**D** and **E**) Networks of genes significantly up-regulated (D) or downregulated (E) by IL-10 signalling blockade (aIL-10R) in unstimulated (black), LPS stimulated (red), L18MDP stimulated (green) and αCD3/αCD28 stimulated (blue) patient samples (fold change > 1.5; node size and edge thickness are proportional to the fold change).

Supplementary Figure 2: Patients with IBD were differentiated clinically based on endoscopy and/or inflammatory activity by blood biomarker and/or symptoms into active versus non-active disease subgroups. (**A**) Correlation matrix of patient characteristics, full blood count markers, IL-23 protein in PBMC culture supernatants (16 hours stimulation) and *IL23A* expression in PBMC from patients with IBD as assessed by qPCR (16 hours stimulation) (n≥28). Only pearson correlations with pvalues<0.01 were colour coded. (**B**) IL-23 protein in 16 hours PBMC culture supernatants from patients with IBD (n≥28; Mean+SD; Mann-Whitney test). (**C**) Relative (*RPLPO*) *IL23A* expression in PBMC from patients with IBD as assessed by qPCR (n≥36; Mean+SD; Mann-Whitney test). (**D**) Normalised expression of *IL23A* in PBMC from patients with IBD as assessed by microarray (n≥35; Mean+SD; Mann-Whitney test).

Supplementary Figure 3: (**A**) Contour plot presentation of IL-23p19-, IL-12p40- and IL-10 producing live leukocytes and CD14 surface expression measured at 16 hours post stimulation in HD PBMC. (**B**) Frequencies of IL-12p40^{+}IL-23p19^{+}, IL-10^{+} and IL-12p40^{+}IL-23p19^{-} CD14^{+} and CD14^{-} cells in HD total live leukocytes (n=26; Mean±SEM; Mann-Whitney test). (**C**) Frequencies of IL-12p40^{+}IL-23p19^{+}, IL-10^{+} and IL-12p40^{+}IL-23p19^{-} of HD CD14^{+} cells (n=26; Mean±SEM; Mann-Whitney test).

Supplementary Figure 4: (**A**) CD14^{+} cell frequencies in total leukocytes following negative or positive selection by magnetic beads, or in PBMC (n≥4) as assessed by flow cytometry. (**B**) Frequencies of monocyte subsets in CD14^{+} cells following negative or positive isolation using magnetic beads or in PBMC as defined by surface expression of CD14, CCR2, and CD16 (n≥4).

(**C**) Dot plot presentation of CCR2 and CD16 expression in $CD14^+$ cells following positive selection using magnetic beads and PBMC.

Supplementary Figure 5: Heatmap of cluster marker genes for cell-cycle corrected analysis of Unstimulated, LPS-stimulated and LPS + anti-IL-10R-stimulated conditions (donor 1).

Supplementary Figure 6: Single cell genomics identifies transcriptionally distinct monocyte clusters following combined LPS + anti-IL-10R stimulation. (**A**) Selected overrepresented GO biological process gene sets of the 8 identified monocyte clusters across stimulation conditions (Unstimulated, LPS-stimulated and LPS + anti-IL-10R-stimulated). **(B)** The dendrogram shows the hierarchical relationship between the 8 identified monocyte clusters across stimulation conditions (Unstimulated, LPS-stimulated and LPS + anti-IL-10R-stimulated). (**C**) Heatmap of cluster marker genes for cell-cycle corrected analysis of the LPS + anti-IL-10R condition alone (donors 1 and 2). Cluster 1 corresponds to cluster 3 in the aligned analysis (Interferon-induced monocytes), cluster 2 to cluster 4 in the aligned analysis ($IL\text{-}23^+$ inflammatory monocytes) and cluster 3 to cluster 6 in the aligned analysis ($MRC1^+$ microbicidial monocyte subpopulations).

Supplementary Figure 7: *IL23A* and *IL10* gene expression positively correlate under settings of LPS stimulation and IL-10 signalling blockade and monocytes produce cytokines with distinct kinetics. (**A**) RT-qPCR based DDCt correlation analysis of *IL10* and *IL23A* gene expression in unstimulated, anti-IL-10R (10 µg/mL), LPS (200 ng/mL), and combined LPS and anti-IL-10R-stimulated PBMC (n=40). (**B**) PBMC were stimulated for up to 66 hours with combinations of LPS and anti-IL-10R. The frequencies of cytokine producing live $CD14^+$ cells were assessed by surface and intracellular staining at different time points, as indicated (n≥4; Mean±SEM; Kruskal-Wallis test; BH-adjusted pvalues). (**C**) ELISpot single staining controls. Monocytes were cultured for 16 hours in the presence of indicated stimuli. Dual-colour ELISpot images were obtained from plates coated with both anti-IL-10 and anti-IL-23 capture antibodies (first row, blue, red and violet/brown spots), anti-IL-10 capture antibodies only (second row, blue spots only) and anti-IL-23 capture antibodies only (third row, red spots only). The results from monocytes of one healthy donor out of two analysed donors are shown.

Supplementary Figure 8: Identification of modules of co-expressed genes in patient biopsies from the paediatric RISK cohort. (**A**) The main heatmap shows expression of the eigen-genes of the n=22 identified modules of genes. Patients (columns) are hierarchically clustered by expression of the module eigen-genes. The heat map is annotated with (i) patient diagnosis and disease subtype (top panels), (ii) expression of key cytokines (upper heatmap), (iii) correlation of the eigen-genes with diagnosis of Crohn's disease, *IL23A* expression and control status (left panel) and (iv) the numbers

of genes within each of the modules (right panel). Modules were named according to enrichments for gene ontology and cell type genesets (Supplementary Table 4). (**B**) The heatmap shows the correlation of the RISK WGCNA modules (y-axis) with genes and traits of interest (x-axis).

Supplementary Figure 9: Characterisation of the RISK WGCNA modules. (**A**) The dotplot shows the enrichment of selected biological process gene ontology categories and KEGG pathways in the WGCNA modules that were correlated with diagnosis of Crohn's disease and/or *IL23A* expression (one-sided Fisher tests, BH-adjusted p<0.05, pvalues adjusted separately for GO categories and KEGG pathways). (**B**) The frequency (y axis) of IBD GWAS-associated genes in the different RISK WGCNA modules (x axis). Genetically associated genes were sourced from Ellinghaus et. al.[67] or de Lange et al.[5] (see methods) BH-adjusted pvalues and odds ratios are given for significant enrichments (red text).

Supplementary Figure 10: Ability of inflammatory monocyte signature genes to predict inflammatory bowel disease and non-response to anti-TNF therapy in three patient cohorts. Comparison of the ability of the identified subsets of monocyte genes (see Figure 4) to predict diagnosis of inflammatory bowel disease and TNF non-response in the RISK (GSE57945), Janssen (GSE12251) and Arijs (GSE16879) cohorts. (**A**) Predictions in Crohn's disease with data from the RISK and Arijs cohorts. (**B**) Predictions in ulcerative colitis with data from the Janssen and Arijs cohorts. The panels from the RISK and Janssen cohorts in (A) and (B) are reproduced from Figure 4. The dashed lines indicate random classifier performance. (**C**) The heatmap shows the AUPRC values of the individual genes for the predictions shown in (A) and (B). (**D**) The heatmap shows the ranks of the predictions for the individual genes. Genes in panels (C) and (D) are ordered by the mean of the AUPRC an AUPRC rank respectively. AUPRC: area under precision recall curve.

Supplementary Figure 11: (**A**) Frequencies of live cells following stimulation of $CD14^+$ monocytes under indicated conditions for 16 hours as assayed by fixable viability dye live/dead staining and flow cytometry (n=6). (**B**) Representative dot plot presentations of live/dead cell discrimination in monocytes following 16 hours stimulation under indicated conditions and STAT3 phosphorylation in response to IL-10 stimulation in the live cells fraction (15 minutes, 50 ng/mL).

Supplementary Figure 12: IL-1α and IL-1β signalling are essential for inducing monocyte IL-23 production. PBMC from patients with IBD (n≥4) were stimulated for 16 hours with combinations of LPS and αIL-10R in the presence of indicated exogenous human recombinant cytokine, cytokine receptor blockade or cytokine blockade. (**A**) The frequencies of cytokine producing live $CD14^+$ cells were assessed by intracellular staining. IL-12p40$^+$IL-23p19$^+$ cell frequencies measured under diverse stimulation conditions (Wilcoxon test; 95% CI). (**B**) Representative dot plot showing

intracellular IL-12p40 and IL-23p19 in $CD14^+$ monocytes of one healthy donor. (**C**) RT-qPCR analysis of healthy donor PBMC stimulated with combinations of LPS, anti-IL-10R, anti-IL-1R1 and IFN-g for 16 hours (n=6; Mean±SD; One-way ANOVA; BH-adjusted pvalues). (**D**) Frequencies of $IL\text{-}12p40^+IL\text{-}23p19^+$ live $CD14^+$ monocytes after 16 hours stimulation with combinations of LPS (200 ng/mL), anti-IL-10R (10 µg/mL) and IL-1β (10 and 50 ng/mL) (n=4).

Supplementary Figure 13: Monocyte IL-23 production induced by stimulation with whole bacteria is dependent on IL-1R1 signalling. (**A**) $IL\text{-}12p40^+IL\text{-}23p19^+$ live $CD14^+$ cells frequencies as assessed by intracellular staining measured in indicated conditions at 16 hours following stimulation (n=8; Mean±SEM; Kruskal-Wallis test; BH-adjusted pvalues). (**B**) Representative dot plots showing intracellular IL-12p40, IL-23p19 and IL-10 in healthy donor-derived live $CD14^+$ monocytes.

SUPPLEMENTARY METHODS

Human samples, cell isolation and cell culture:

Patients with IBD and controls were recruited via the Oxford IBD cohort and Gastrointestinal biobank (REC 11/YH/0020 and 16/YH/0247). All patients with IBD and healthy volunteers provided written informed consent. Peripheral blood mononuclear cells (PBMC) were purified using Ficoll-Paque density gradient purification. For stimulation assays, $0.5x10^6$ -$1x10^6$ PBMC or MACS-purified $CD14^+$ monocytes (Mitenyi Biotec) were cultured in 200 µl medium in duplicates in round bottom 96-well plates. For detailed description of cell culture and stimulation, including whole bacteria preparation for stimulation, see the respective subsection of the supplementary methods.

Patient cohort - RISK cohort and outcome classification:

The RISK study is an observational prospective cohort study with the aim to identify risk factors that predict complicated course in paediatric patients with Crohn's disease[26]. The RISK study recruited treatment-naive patients with a suspected diagnosis of Crohn's disease. The Paris modification of the Montreal classification were used to classify patients according to disease behaviour (non-complicated B1 disease (non-stricturing, non-penetrating disease); complicated disease, composed of B2 (stricturing) and/or B3 (penetrating) behaviour) as well as disease location (L1, ileal only, L2, colonic only, L3, ileocolonic and L4, upper gastrointestinal tract). 322 samples were investigated with ileal RNA-seq. Individuals without ileal inflammation were classified as non-IBD controls. Patients with Crohn's disease were followed over a period of 3 years. Patients were largely of European (85.7%) and African (4.1%) ancestry.

Patients with ulcerative colitis Montreal classification: disease extend : E1=3.3%; E2=19.7%; E3=62.3%; Inactive=14.7%; Location-specific involvement (mic=microscopic inflammation; mac=macroscopic inflammation): cecum: not inflamed=37.3%; mic=19.7%; mac=42.7% | ascending colon: not inflamed=36.1%; mic=16.4%; mac=47.5% | transverse colon: not inflamed=27.9%; mic=9.8%; mac=62.3% | descending colon: not inflamed=16.4%; mic=14.8%; mac=68.8% | sigmoid: not inflamed=18.0%; mic=4.9%; mac=77.1% | rectum: not inflamed=11.5%; mic=6.6%; mac=81.9%)

Cell culture and stimulation:

For stimulation assays, $0.5x10^6$ -$1x10^6$ PBMC or MACS-purified $CD14^+$ monocytes (Mitenyi Biotec) were cultured in 200 µl medium in duplicates in round bottom 96-well plates and exposed to ultrapure 200 ng/mL LPS (Enzo Life Sciences; Cat.# ALX-581-008), 200 ng/mL L18MDP (InvivoGen) 10 µg/mL anti-IL-10R (Biolegend; clone: 3F9), anti-CD3/anti-CD28 beads (Invitrogen) or Staphylococcal enterotoxin B (SEB; Sigma) for the indicated time in complete RPMI with L-glutamine (Sigma) supplemented with 10 % human serum (Sigma; Cat.# H4522), non-essential amino acids (Gibco); 1 mM Sodium-Pyruvate (Gibco) and 100 U/mL penicillin and 10 µg/mL streptomycin (Sigma). Supernatants were collected and stored at -80 °C for the quantification of cytokine production. The following neutralizing antibodies or receptor blocking antibodies were used (all 10 µg/mL): anti-IL-24 (R&D; Cat.# AF1965; Polyclonal Goat IgG), anti-IL-12p70 (R&D; Cat.# MAB219; clone 24910), anti-IL-19 (R&D; Cat.# MAB10351; clone 152107), anti-GM-CSF (R&D; Cat.# MAB215; clone 3209), anti-IL-1R1 (R&D; Cat.# AF269; polyclonal Goat IgG), anti-IL-1α/IL-1F1 (R&D; Cat.# AF-200-NA; polyclonal Goat IgG), anti-IL-1β/IL-1F2 (R&D; Cat.# MAB201; clone: 8516), anti-IL-6R (Tocilizumab, Actemra®, Roche,), anti-TNFα (Infliximab, REMICADE®, Janssen), anti-IFNα/βR2 (Millipore; Cat.# MAB1155; clone: MMHAR-2), anti-IL-10R (Biolegend; Cat.# 308807; clone: 3F9), anti-IL-10 (R&D; Cat.# MAB217; clone: 23738), anti-IFN-γ (Biolegend; Cat.# 506513; clone: B27), anti-IL-36γ (R&D; Cat.# MAB2320; clone: 278706). The following recombinant human cytokines were used (all 10 ng/mL): IL-1β (Preprotech), IL-6 (Preprotech), IL-10 (Preprotech), IL-11 (R&D), IL-12 (Preprotech), IL-17A (Preprotech), IL-18 (R&D), IL-23 (Preprotech), GM-CSF (Preprotech), IFN-γ (Preprotech), TNFα (Preprotech).

Whole bacteria preparation and stimulation:

Both *Salmonella enterica serovar Typhimurium* (ST) (NCTC 12023)[55] and *Escherichia coli* Nissle 1917[56] (EcN) were grown overnight in LB (Sigma) and the O.D was measured on the following day

at 650nm. Aliquots corresponding to 10^9 particles of bacterial were centrifuged at 6000 rpm for 5 minutes and the pellet was washed twice in the fresh LB then twice in PBS (Sigma). Cells were then heat-killed at 65°C for 30 minutes and stored in aliquots at -20°C till further use. For monocyte stimulation assays, $0.1x10^6$ MACS-purified $CD14^+$ monocytes (Mitenyi Biotec) were cultured in 150 µl medium in duplicates in round bottom 96-well plates and exposed to combinations of ST (monocyte to bacteria ratio = 1:2), EcN (monocyte to bacteria ratio = 1:2), ultrapure 200 ng/mL LPS (Enzo Life Sciences; Cat.# ALX-581-008), 10 µg/mL anti-IL-10R (Biolegend; clone: 3F9), 10 µg/mL anti-IL-10 (R&D; Cat.# MAB217; clone: 23738), 10 µg/mL anti-IL-1R1 (R&D; Cat.# AF269; polyclonal Goat IgG) for 16 hours (the last 4 hours of culture in the presence of BFA (Sigma) in the case of intracellular staining) in complete RPMI with L-glutamine (Sigma) supplemented with 10 % human serum (Sigma; Cat.H4522), non-essential amino acids (Gibco); 1 mM Sodium-Pyruvate (Gibco) and 100 U/mL penicillin and 10 µg/mL streptomycin (Sigma).

Protein level analysis:

Cytokine levels in the supernatants were assayed using the Milliplex human cytokine/chemokine magnetic bead 41-plex panel (Milliplex Billerica, MA, USA; HCYTOMAG-60K-PX41) and acquired on a Luminex LX200 flow reader. IL-23p19 cytokine level was assayed using a MSD (Meso Scale Discovery) in-house assay. In short, an IL-23p-19 selective antibody (Ab1, clone #32) was biotinylated following the Biotin, EZ-Link™ NHS-PEG4-Biotin instructions (ThemoScientific, Cat.# 2161299). For detection, an anti-p40 antibody (Ab-2, clone #42) was sulfotaged according to manufactures instructions (MSD Gold Sulfo-Tag NHS; Cat.# R91A0-1 and MSD Gold Sulfo-Tag NHS-Ester Conjugation Packs; Cat.# R31AA-1). MSD Streptavidin Gold plates (MSD; Cat.# L15SA-1) were washed, blocked, coated with biotinylated Ab-1, and washed according the manufacture instruction. 50 µL of supernatant or standard were added to the plate and incubated overnight at 4 °C while shaking. Plates were washed 3 times and detection antibody was added and incubated for 2 hrs while shaking at room temperature. Plates were washed and read buffer added. Plates were read with a MSD reader Quick Plex S120.

Surface marker analyses by flow cytometry:

The expression of cell surface markers was analysed by staining for 15 min at room temperature (RT) in PBS supplemented with 0.5% (v/v) human serum (FACS buffer). To allow exclusion of dead cells, cells were stained using Fixable Viability Dye eFluor® 780 (eBioscience). Following fluorophore-conjugated antibodies were used for analysis (supplier, clone): anti-CD3 (BD Biosciences; UCHT1), anti-CD4 (Biolegend; RPA-T4), anti-CD8 (Biolegend; SK1), anti-CD14

(Biolegend; M5E2), anti-CD16 (Biolegend; 3G8), anti-CD19 (BD Biosciences; SJ25C1), anti-CD25 (Biolegend; MA-A251), anti-CD45RA (Biolegend; HI100), anti-CD56 (BD Biosciences; NCAM16.2), anti-HLA-DR (Biolegend; L243), anti-CCR2 (Biolegend; K036C2). For combined analysis of surface marker and intracellular cytokines, surface-stained cells where subsequently stained as described in the section Intracellular cytokine staining. Data were acquired on a LSRII flow cytometer (BD Biosciences).

Intracellular cytokine staining (ICCS):

Cells were stimulated in the presence of brefeldin A (BFA, 10 μg/mL, all from Sigma) for the final 4 hours of cell culture. Cells were fixed and permeabilised with Cytofix/Cytoperm (BD Biosciences) according to the manufacturer's instructions. To exclude dead cells from the analysis, cells were stained prior to fixation using Fixable Viability Dye eFluor® 780 (eBioscience). Following fluorophore-conjugated anti-cytokine antibodies were used for analysis (supplier; clone): anti-IL-1α (Biolegend; 364-3B3-14), anti-IL-1β (Biolegend; H1b-98), anti-IL-4 (BD Biosciences; MP4-25D2), anti-IL-6 (eBioscience; MQ2-13A5), anti-IL-10 (Biolegend; JES3-9D7), anti-IL-13 (eBioscience: 85BRD), anti-IL-17A (eBioscience; eBio64DEC17), anti-IL-23p19 (eBioscience; 23dcdp), anti-GM-CSF (BD Biosciences; BVD2-21C11), anti-IFN-γ (eBioscience; 4S.B3), anti-TNF (Biolegend: MAb11). For the detection of IL-12p40 the combination of biotin-conjugated anti-IL-12p40 (BD Biosciences; C8.6) and streptavidin STV-CF594 (BD Biosciences; Cat.# 562318) were used. Data were acquired on a LSRII flow cytometer (BD Biosciences).

Monocyte cluster validation and tSNE analysis of flow cytometry data:

For the validation of scRNA-seq-identified monocyte clusters at the protein level, cells were stained for the following marker (supplier; clone): anti-CD14 (Biolegend; M5E2), anti-HLA-DR (Biolegend; L243), anti-CCL20/MIP-3 alpha (R&D; 67310), anti-CCL20/MIP-3 alpha (R&D; 114906), anti-IL-23p19 (eBioscience; 23dcdp), anti-CCL2 (eBioscience; 5D3-F7), anti-RPS6 (R&D; 522731), anti-SPINK1 (R&D; 839305), anti-IDO-1 (eBioscience; Eyedio), EBI3 (eBioscience; ebic6), anti-S100A8 (Invitrogen: CF-145), anti-S100A9 (Biolegend, MRP-14), anti-TNF (Biolegend: MAb11). Unconjugated antibodies targeting RPS6 and SPINK were conjugated in house using the Expedeon (Innova Biosciences) Lightning–Link PE/Texas Red or DyLight 405 antibody labelling kit according to the manufacturer's instructions, respectively. t-Distributed Stochastic Neighbor Embedding (TSNE)-based analysis was executed on FCS files compensated for spillover between channels and gated on live $CD3^{-}CD19^{-}CD56^{-}CD14^{+}$ single cells, down-sampled to 3.000 cells per sample. A single FCS file was generated by concatenating individual samples FCS files prior to tSNE unsupervised analysis using the FlowJo (Treestar) tSNE plugin[57 58]

based on cluster defining genes. The following settings were used: Iterations = 1000; Perplexity = 200; Eta = 20; Theta = 0.5. and included the following parameters: CD14, HLA-DR, CCL2, CCL20, IL-23p19, IDO-1, RPS6, SPINK1, S100A8 and TNF. IL-23^{+} inflammatory monocytes were defined by combined IL-23p19, CCL20 and TNF expression, IFN-induced monocytes were defined by combined HLA-DR and IDO-1expression; microbicidal monocytes were defined by combined CCL2, S100A8 and CD14 expression and apoptotic monocytes were identified by combined RPS6 and SPINK-1expression). Each individual analysis was performed on samples that were stained and acquired (LSRII (BD Biosciences)) on the same day.

Analysis of cytokine-induced STAT3 phosphorylation by flow cytometry:

For the analysis of IL-10 responsiveness in stimulated monocytes, cells were washed (2 times) in RPMI (Sigma) and incubated in RPMI for 2 hours. Following incubation, cells were washed (2 times) in RPMI and stimulated for 15 minutes in complete RPMI with 50 ng/mL recombinant human IL-10 (Preprotech). Cells were then stained in PBS (Sigma) on ice with Fixable Viability Dye eFluor® 780 (eBioscience). Subsequently, cells were fixed in pre-warmed (37°C) BD Cytofix for 10 minutes (BD biosciences) at 37°C. After fixation, cells were permeabilised on ice with ice-cold Perm Buffer III (BD biosciences) and stained with anti-pSTAT3 (pY705)-Alexa Fluor 647 (4/P-STAT3, BD Phosflow) for 1 h at room temperature before acquiring on a LSRII flow cytometer (BD Biosciences).

IL-10 and IL-23 ELISpot assay:

ELISpot plates (Merck; Cat.#MSHAS4510) were incubated with sterile PBS (Gibco) and coated over night at 4°C with anti-IL-12/IL-23p40 (Mabtech; Cat.# MT86/221; 10 µg/mL in PBS) and anti-IL-10 (Mabtech; Cat.# 9D7; 15 µg/mL in PBS) antibodies using 100 µL/well. On the next day plates were flicked and blocked using 100 µL/well 1x sterile PBS (Gibco) supplemented with 10% FCS (Sigma) for 2 hours at 37°C. Following, the buffer was removed and MACS-purified monocytes were plated at 2-fold concentration steps ranging from 6250 to 50.000 cells/well in complete RPMI medium supplemented with 10% FCS. Stimulation and incubation was performed over night at 37°C and 5% CO2 using combinations of LPS (200 ng/mL) and IL-10R blocking antibody (10 µg/mL). Following 16 hours incubation, cells were removed by flicking the plate and wells were washed extensively 10 times, each 1 minute incubation, with 200 µL washing buffer (0.1% Tween20 (Sigma) in PBS (Gibco). Secondary antibodies were diluted in PBS supplemented with 0.5% FCS (anti-IL-23p19 (Mabtech; Cat.#/clone: MT155-HRP; 1 µg/mL) and anti-IL-10-biotin (Mabtech; Cat.#/clone: 12G8: 1 µg/mL). 100 µl/well antibody mixture was added to each

well and incubation was performed for 2 hours at 37°C. Plates were then washed extensively 10 times, each 1 minute incubation. For the development of spots first 100 µL/well substrate solution (BCIP/NBT; Sigma) was added and development continued until distinct blue/grey spots emerged (approximately 5 minutes). Plates were again extensively washed 10 times, each 1 minute incubation, with 200 µL washing buffer. Then 100 µL/well substrate solution (AEC; BD Pharmingen) was added to each well and development was continued until distinct red spots emerged (approximately 5 minutes). The anti-IL-10 clone 12G8 was conjugated with biotin (Novus Biologicals Lightning-Link Rapid Type A Biotin Antibody Labeling Kit). Plates were left to dry in the dark and images were acquired using an AID EliSpot Reader Systems. Acquired pictures were manually inspected and quantified using Fiji/ImageJ software[59]. Images shown in Figure 3D and Supplementary Fig. 5C were adjusted using the Fiji/ImageJ background correction plugin for improved presentation quality (Rolling ball radius = 50.0 pixels).

Generation and pre-processing of single-cell RNA-sequencing data:

$CD14^+$ monocytes were purified from PBMC by MACS positive selection (Mitenyi Biotec) according to the manufacturers' instructions from 2 human donors. The purity of sorting and viability was assessed by surface staining and flow cytometry (anti-CD14 (Biolegend; M5E2); Fixable Viability Dye eFluor® 780 (eBioscience). Cells were plated in 96-well U-bottom plates at a cell density of $0.5x10^6$ cells/well in 200 µl complete RPMI and were left unstimulated or exposed to combinations of ultrapure 200 ng/mL LPS (Enzo Life Sciences; Cat.# ALX-581-008) and 10 µg/mL anti-IL-10R (Biolegend; clone: 3F9) for 16 hours. For droplet-based single cell RNA-sequencing analysis cells were collected in RPMI with L-glutamine (Sigma) supplemented with 0.5% human serum (Sigma; Cat.# H4522) and the cell number was adjusted to 1000 cells/µl. Single cell suspensions were kept on ice, washed in PBS with 0.04% BSA and resuspended. 10.000 single cells/channel were captured in droplets on Chromium 10x Genomics platform (less than one hour following the termination of stimulation). Library generation for 10x Genomics v2 chemistry was performed following the Chromium Single Cell 3′ Reagents Kits User Guide (CG00052). Quantification of library was performed using an Agilent Bioanalyzer and Bioanalyzer High Sensitivity DNA Reagents (Cat.# 5067-4627). Single-cell RNA-sequencing libraries were generated using the 10x Genomics Single Cell 3' Solution (version 2) kit and sequenced to a minimum mean depth of 44.3k reads/cell (Illumina HiSeq 4000). An average of 2850 cells/per sample and 1150 genes/cell were recovered. Data analysis was performed using Python3 pipelines (https://github.com/sansomlab/tenx) written using the CGAT-core library #https://doi.org/10.12688/f1000research.18674.1. Read mapping, quantitation and aggregation of sample count matrices was performed with the 10x Genomics Cell Ranger pipeline (version 2.1.1)

and version 1.2.0 (GRCh38) reference sequences. No normalisation was applied during the aggregation step. Cells with barcodes common to samples sequenced on the same lane(s) were removed from the analysis to avoid issues associated with index hopping. Aggregated count matrices were randomly down-sampled in order to normalise the median number of UMIs per-cell between the samples ("downsampleMatrix" function from the DropletUtils R package). Down-sampling was performed separately for the within-donor cross-condition analysis (Figure 2) and the cross-donor analysis of LPS + anti-IL10R stimulated monocytes (Supplementary Fig. 3B).

Cross-condition analysis of single-cell RNA-sequencing data from a single donor:

The dataset was filtered to remove cells with <500 genes, >5% mitochondrial reads per cell or that expressed lymphocyte markers (*CD3*, *CD79A* or *CD79B*, n=33 cells) leaving a total of n=2420 unstimulated, n=3149 LPS stimulated and n=2273 LPS + anti-IL-10R stimulated monocytes. Per-cell UMI counts were then normalised, scaled and the effects of total UMI counts, percentage of mitochondrial counts and cell cycle ("all" effects; using known G2 and S phase associated genes[60]) regressed out with the Seurat R package (version 2.3.4). Significantly variable genes (n=1164) were selected using the "trendVar" function from the R Bioconductor package Scran (minimum mean log-expression 0.05, BH adjusted p-value < 0.05). These genes were used as input for principal component analysis (PCA), and significant PCs (n=33) identified using Seurat ("JackStraw" test, BH adjusted p < 0.05). These principle components were used as input for alignment with Harmony databaseFigure 2E) comprised of those from Martin et. al. Figure 2D and Fgure 5D and Smillie et. al. Figure 1G anFigure 3E

Comparison of LPS + anti-IL-10R single-cell RNA-sequencing data from two donors.

The dataset was filtered as for the cross-condition analysis (removing n=23 contaminating lymphocytes) and an equal number (n=2280) of cells randomly sub-sampled from each donor for further analysis. Normalisation and scaling (including regression of cell-cycle effects), identification of variable genes (n=849), identification of significant PCs (n=35) and clustering (resolution=0.6) was performed as described above (no alignment performed).

Identification and characterisation of gene co-expression modules in the RISK cohort:

RPKM expression values for the RISK cohort[26 63] were retrieved from GEO (GSE57945), upper-quartile normalised and log2(n+1) transformed. Prior to WGCNA analysis the dataset was filtered to retain (n=13,850) genes that had a transformed expression value ≥ 1.5 in > 10% of the patients.

The WGCNA R package (version 1.66) was then applied as follows (i) data was cleaned using the “goodSamplesGenes” function (with parameters: minFraction=0.5, minNsamples=4, minNGenes=4, minRelativeWeight=0.1), (ii) outlying samples (n=11) were identified and removed using the “cutreeStatic” function (with parameters: cutHeight=110, minSize=20), (iii) adjacencies calculated with the “adjacency” function (parameters: type=signed_hybrid, power=5, corFnc=cor, corOptions use=p and method=spearman”), (iv) the topological overall matrix computed with the “TOMsimilarity” function (parameter TOMType=unsigned), (v) a dynamic tree cut performed with function “cutreeDynamic” (parameter deepSplit=2, pamRespectsDendro=FALSE, minClusterSize=30) and (vi) modules merged with the “mergeCloseModules” function (parameter cutHeight=0.25). Genes assigned to modules were subject to geneset over-representation analysis as described for the single-cell RNA-sequencing analysis with gene categories obtained from the Gene Ontology (GO) databaseG pathways genesllinghau*et. al.* (Ellinghaus Supplementary Table 3a, n=156 genes) or by de Lnge *et. al.* (de Lange Supplementary Table 2). The associated genes from de Lange *et. al.* were conservatively filtered to retain only “implicated” genes or genes that originate from single-gene loci (n=89 genes, 48 of which overlap with the Ellinghaus *et. al.* set).

Identification and characterisation of an IL-10-responsive monocyte gene signature:

The IL-10 regulated inflammatory monocyte gene signature (Figure 4E) was constructed from (i) genes that showed ≥ 3-fold higher expression in at least one of the three LPS + aIL10R clusters than was found in any of the unstimulated or LPS-stimulated clusters (single-cell RNA-sequencing analysis, Figure 2A), (ii) genes that showed significantly higher expression in the $CD14^+$ positive PBMC fraction upon anti-IL-10R + LPS stimulation versus LPS stimulation alone (n=1, IL1B, Supplementary Fig. 5), and (iii) two genes from the literature: CD14, a well-established monocyte type cell marker and OSM which is known to be expressed by inflammatory monocytes in human IBD and to drive colitis in the *Hh* + anti-IL-10R model[18]. The predictive ability of genes in the IL-10 regulated inflammatory monocyte signature was investigated in RNA-seq data from the RISK cohort and Affymetrix microarray data from the Janssen (UC-cohort GSE12251, Figure 4F). The UC-cohort GSE12251 includes data from patients with active UC that had a total Mayo score between 6-12 and an endoscopic sub-score of at least 2. The response to infliximab was defined as endoscopic and histologic healing in patients that underwent a second flex sigmoidoscopy with rectal biopsies 4 weeks after the first infliximab infusion in case of a single infusion and at 6 weeks if they received a loading dose of infliximab at weeks 0, 2 and 6. Microarray datasets from the Janssen (GSE12251) and Arijs (GSE168)

Real-time PCR:

Real-time PCRs were performed in 96-well plates using the PrecisionPLUS qPCR Mater Mix (Primer Design) and the CFX96 Touch Real-Time PCR Detection System machine (BIO-RAD). The expression of transcripts was normalized to expression of Large Ribosomal Protein (*RPLPO*). Data analysis was performed using the Cycles threshold (ΔΔCt) method and expressed as mRNA relative expression ΔΔCt. The following TaqMan probes (Applied Biosystems) were used for qPCR analysis: *IL23A* (Hs00900828_g1), *IFNG* (Hs00989291_m1), *RPLPO* (Hs99999902_m1).

Gene array expression and statistical analysis:

Background correction and quantile normalization of the gene expression data obtained from the Affymetrix Human Transcriptome Array 2.0 (HTA2) platform were performed by the RMA method[16]. Each gene's expression was defined at the level of the "transcript cluster", which is the collection all probesets on the HTA2 platform that are located within a gene's annotated position in the genome. These gene positions were defined by the Affymetrix NetAffxTM NA34 release, which is based on the GRCh37 human reference genome. Differential expression at the gene-level was performed using the empirical Bayes method implemented in LIMMA[69] controlling for donor in the model. Differential expression between a given stimulation vs. PBMC control contrast was considered at a Benjamini-Hochberg adjusted p-value < 0.05 and absolute fold change > 2. A heatmap was produced using the heatmap.2 function from the gplots package (R-3.4.2) to visualise the union of genes that were regulated in each stimulation vs. PBMC control contrast.

Mathematical modelling of cytokine interactions.

We define a system of ordinary differential equations of the form $\frac{dx_i}{dt} = s_i \prod_u \left(1 + \alpha_{u,i} x_u\right) \prod_v \left(1 + \beta_{v,i} x_v\right)$, with x_i the concentration of TNF, IL-1α, IL-1β, IL-6, IL-10 or IL-23 over time, $s_i$the contribution of LPS, and $\alpha_{u,i}$ ($\beta_{u,i}$) the positive (negative) effect of cytokine u on the production rate of cytokine i. With additional time and/or dose dependent data, the model could be extended by including saturation of cytokine production (e.g. using Michaelis-Menten or Hill functions) or cytokine degradation. By minimizing the log likelihood, we fit the right-hand side of the equations to 1750 experimental data points, representing cytokine production rates by monocytes 16 hours after LPS stimulation. These rates were determined by intracellular flow cytometry in PBMC derived from healthy individuals, in the presence or absence of anti-IL-10R, and TNF, IL-1β, IL-6, IL-10, IL-23, IFN-γ, anti-IFN-γ, anti-IFNR1, anti-IL-1α, anti-IL-1β, anti-IL-1R1, anti-IL-6R, or anti-TNF (n=3-17). The frequency of cytokine (i.e. TNF, IL-1α, IL-1β, IL-6, IL-10 or IL-23) producing live $CD14^+$ cells multiplied by the mean fluorescence intensity of the respective cytokine producing cell population was taken as a measure of cytokine production

rate. All possible network configurations using a set of edges representing significant (paired Wilcoxon test, p≤0.05) direct cytokine interactions derived from this dataset were considered, and for each number of edges, an optimal model configuration was found. We did not model all 2^{31}-1 possible configurations explicitly, as the vast majority of configurations could be logically excluded based on a small subset of network configuration fits. It was found that all optimal models were nested, i.e. the list of optimal models can be obtained by iteratively adding edges to the model. As additional edges will always improve the model fit, we use the Akaike Information Criterion (AIC) to identify the fourteen-edge model as the model with an optimal balance between edge number and model fit. For two nested models that differ one edge, the AIC is equivalent to a likelihood ratio test (LRT, alpha=0.16) with one degree of freedom. Fitting and parameter identifiability analysis of the fourteen-edge model was done in the Matlab based modelling environment 'Data2Dynamics', using a deterministic trust-region approach combined with a multi-start strategy; data2dynamics.org[70].

Correlation analysis of patients with IBD clinical parameters and PBMC culture IL-23 protein and IL23A mRNA expression:

Protein level data for IL-23 concentrations, relative *IL23A* expression qPCR data, full blood count data from patients with IBD, serum C-reactive protein level, patients with IBD disease activity (differentiated clinically based on endoscopy and/or inflammatory activity by blood biomarker and/or symptoms), diagnosis (CD, UC), patient age, age at diagnosis and gender were analysed with R-4.0.0 and RStudio-1.1.456. The pearson correlation matrix was generated using the stats package 3.6.3 "cor" function. Pvalues were calculated using the corrplot 0.84 package "cor.mtest" function. The correlation plot was generated using the corrplot 0.84 package "corrplot" function. Data were ordered using the "hclust" function (ward.D2). Only pearson correlations with a pvalue<0.01 were colour coded.

Resources for data visualization:

Gene and protein networks were visualized using Cytoscape 3.6.1. http://www.cytoscape.org/[71]. Heatmaps for gene expression data visualisation were generated using the GENE-E or Morpheus tool from the Broad Institute: http://www.broadinstitute.org/cancer/software/ GENE-E/; https://software.broadinstitute.org/morpheus/.

Statistical analysis

Statistical analyses were performed with GraphPad Prism, version 8.0 for Macintosh (GraphPad Software, La Jolla, CA) or Microsoft Excel for Mac, version 15.32. P-values ≤ 0.05 were considered significant and indicated as follows: *P < 0.05; **P < 0.01; ***P < 0.001; ****P < 0.0001. Statistical tests are described in figure legends.

SUPPLEMENTARY REFERENCES

55. Schulthess J, Pandey S, Capitani M, et al. The Short Chain Fatty Acid Butyrate Imprints an Antimicrobial Program in Macrophages. *Immunity* 2019;50(2):432-45 e7. doi: 10.1016/j.immuni.2018.12.018 [published Online First: 2019/01/27]
56. Sonnenborn U. Escherichia coli strain Nissle 1917-from bench to bedside and back: history of a special Escherichia coli strain with probiotic properties. *FEMS Microbiol Lett* 2016;363(19) doi: 10.1093/femsle/fnw212 [published Online First: 2016/09/14]
57. van der Maaten L, Hinton G. Visualizing non-metric similarities in multiple maps. *Mach Learn* 2012;87(1):33-55. doi: 10.1007/s10994-011-5273-4
58. Wallach I, Lilien R. The protein-small-molecule database, a non-redundant structural resource for the analysis of protein-ligand binding. *Bioinformatics* 2009;25(5):615-20. doi: 10.1093/bioinformatics/btp035 [published Online First: 2009/01/21]
59. Schneider CA, Rasband WS, Eliceiri KW. NIH Image to ImageJ: 25 years of image analysis. *Nat Methods* 2012;9(7):671-5. doi: 10.1038/nmeth.2089 [published Online First: 2012/08/30]
60. Dominguez D, Tsai YH, Gomez N, et al. A high-resolution transcriptome map of cell cycle reveals novel connections between periodic genes and cancer. *Cell Res* 2016;26(8):946-62. doi: 10.1038/cr.2016.84 [published Online First: 2016/07/02]
61. Korsunsky I, Millard N, Fan J, et al. Fast, sensitive and accurate integration of single-cell data with Harmony. *Nat Methods* 2019;16(12):1289-96. doi: 10.1038/s41592-019-0619-0 [published Online First: 2019/11/20]
62. Ashburner M, Ball CA, Blake JA, et al. Gene ontology: tool for the unification of biology. The Gene Ontology Consortium. *Nat Genet* 2000;25(1):25-9. doi: 10.1038/75556 [published Online First: 2000/05/10]
63. Haberman Y, Tickle TL, Dexheimer PJ, et al. Pediatric Crohn disease patients exhibit specific ileal transcriptome and microbiome signature. *J Clin Invest* 2014;124(8):3617-33. doi: 10.1172/JCI75436 [published Online First: 2014/07/09]
64. Harris MA, Clark J, Ireland A, et al. The Gene Ontology (GO) database and informatics resource. *Nucleic Acids Res* 2004;32(Database issue):D258-61. doi: 10.1093/nar/gkh036 [published Online First: 2003/12/19]
65. Kanehisa M, Furumichi M, Tanabe M, et al. KEGG: new perspectives on genomes, pathways, diseases and drugs. *Nucleic Acids Res* 2017;45(D1):D353-D61. doi: 10.1093/nar/gkw1092 [published Online First: 2016/12/03]
66. Aran D, Hu Z, Butte AJ. xCell: digitally portraying the tissue cellular heterogeneity landscape. *Genome Biol* 2017;18(1):220. doi: 10.1186/s13059-017-1349-1 [published Online First: 2017/11/17]
67. Ellinghaus D, Jostins L, Spain SL, et al. Analysis of five chronic inflammatory diseases identifies 27 new associations and highlights disease-specific patterns at shared loci. *Nat Genet* 2016;48(5):510-8. doi: 10.1038/ng.3528 [published Online First: 2016/03/15]
68. Arijs I, De Hertogh G, Lemaire K, et al. Mucosal gene expression of antimicrobial peptides in inflammatory bowel disease before and after first infliximab treatment. *PLoS One* 2009;4(11):e7984. doi: 10.1371/journal.pone.0007984 [published Online First: 2009/12/04]

69. Ritchie ME, Phipson B, Wu D, et al. limma powers differential expression analyses for RNA-sequencing and microarray studies. *Nucleic Acids Res* 2015;43(7):e47. doi: 10.1093/nar/gkv007 [published Online First: 2015/01/22]
70. Raue A, Steiert B, Schelker M, et al. Data2Dynamics: a modeling environment tailored to parameter estimation in dynamical systems. *Bioinformatics* 2015;31(21):3558-60. doi: 10.1093/bioinformatics/btv405 [published Online First: 2015/07/05]
71. Shannon P, Markiel A, Ozier O, et al. Cytoscape: a software environment for integrated models of biomolecular interaction networks. *Genome Res* 2003;13(11):2498-504. doi: 10.1101/gr.1239303 [published Online First: 2003/11/05]
