# Supplementary figures and images for: Deconvolution of monocyte responses in inflammatory bowel disease reveals an IL-1 cytokine network that regulates IL-23 in genetic and acquired IL-10 resistance
Source: Gut. 2020 Oct 9;70(6):1023–36. doi: 10.1136/gutjnl-2020-321731 (PMC8108288; doi:10.1136/gutjnl-2020-321731)

Supplementary Figure 1

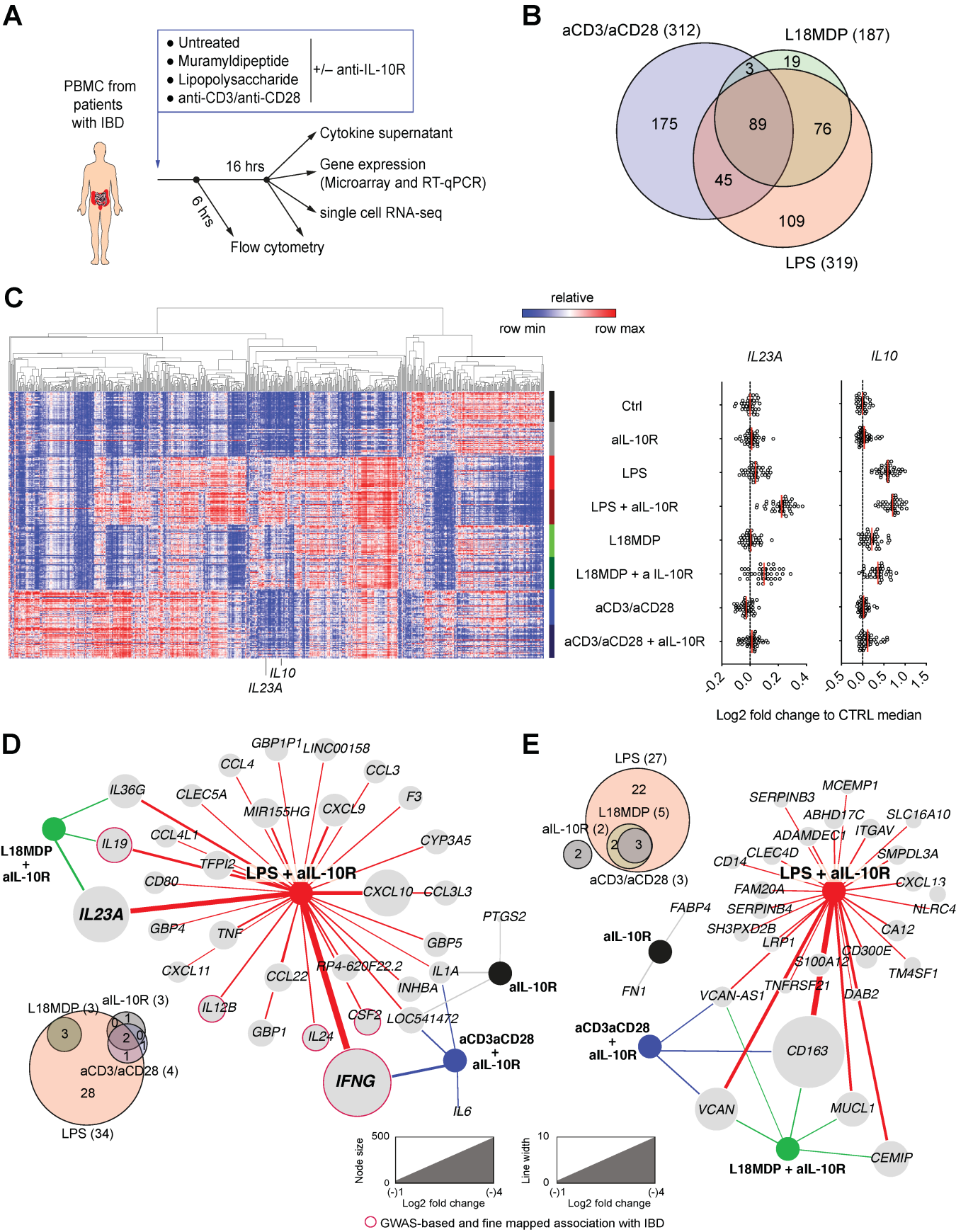

Supplement: Supplementary data [file gutjnl-2020-321731supp002.pdf]

Supplementary Figure 2

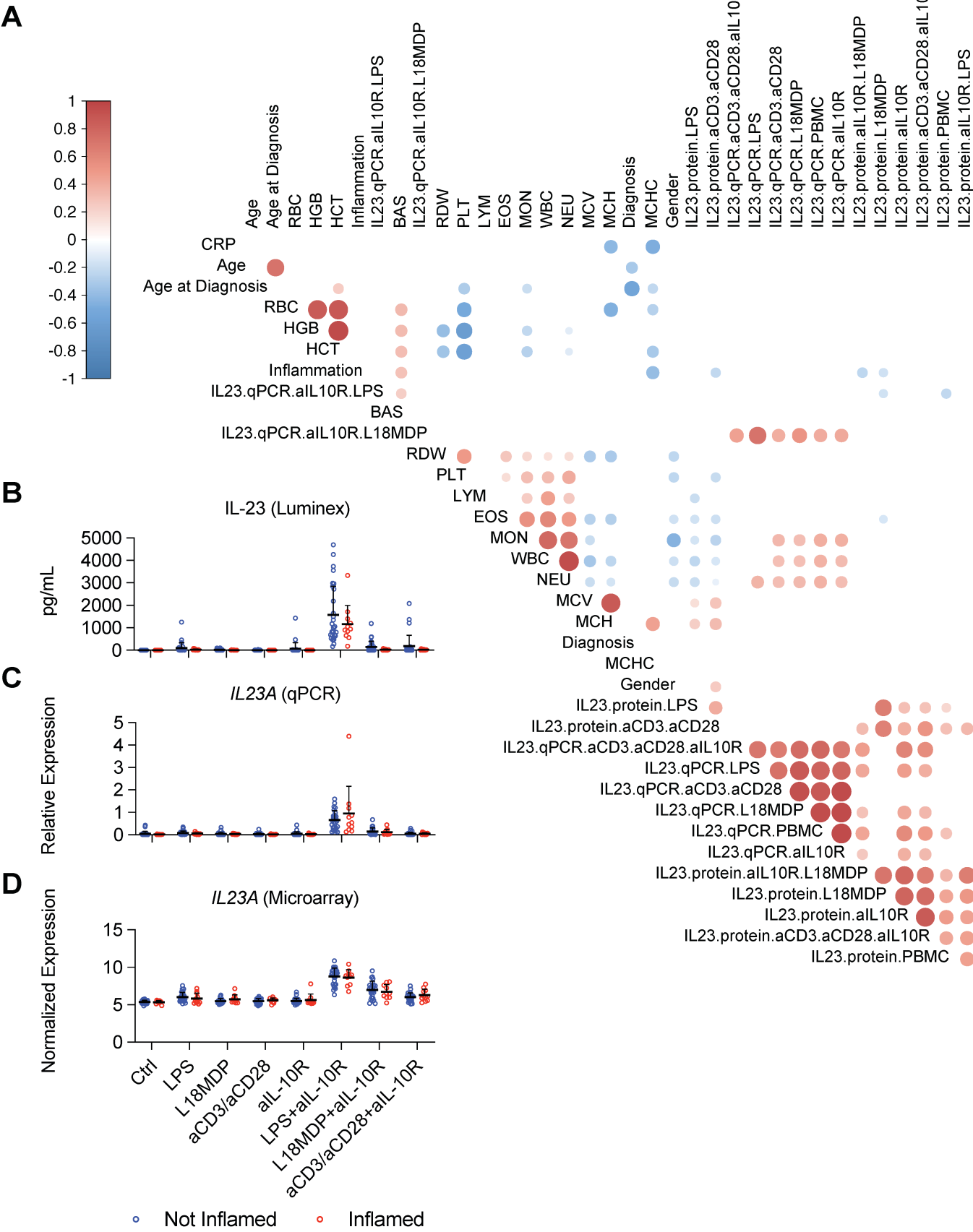

Supplement: Supplementary data [file gutjnl-2020-321731supp006.pdf]

Supplementary Figure 3

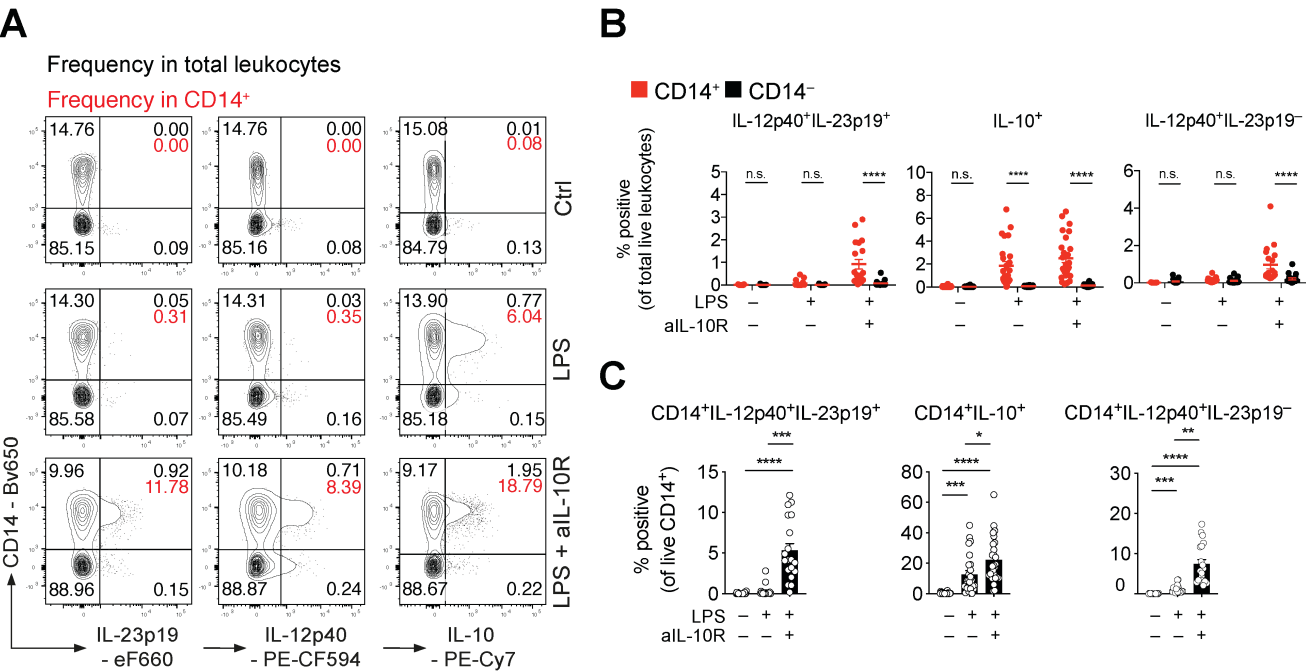

Supplement: Supplementary data [file gutjnl-2020-321731supp007.pdf]

Supplementary Figure 4

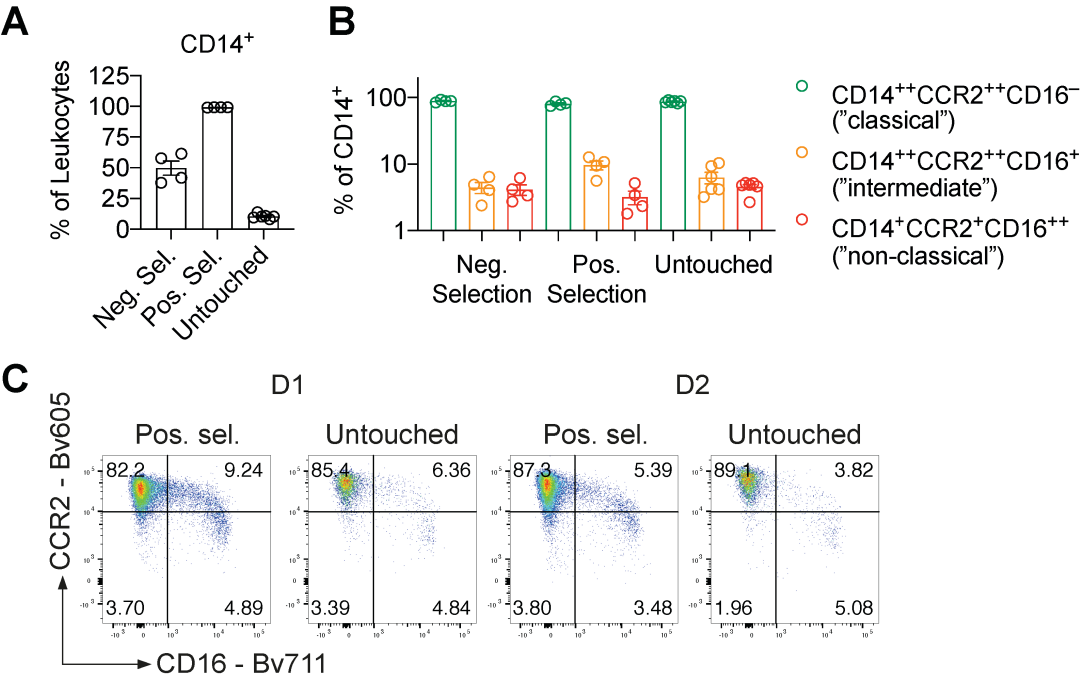

Supplement: Supplementary data [file gutjnl-2020-321731supp008.pdf]

### Supplementary Figure 6

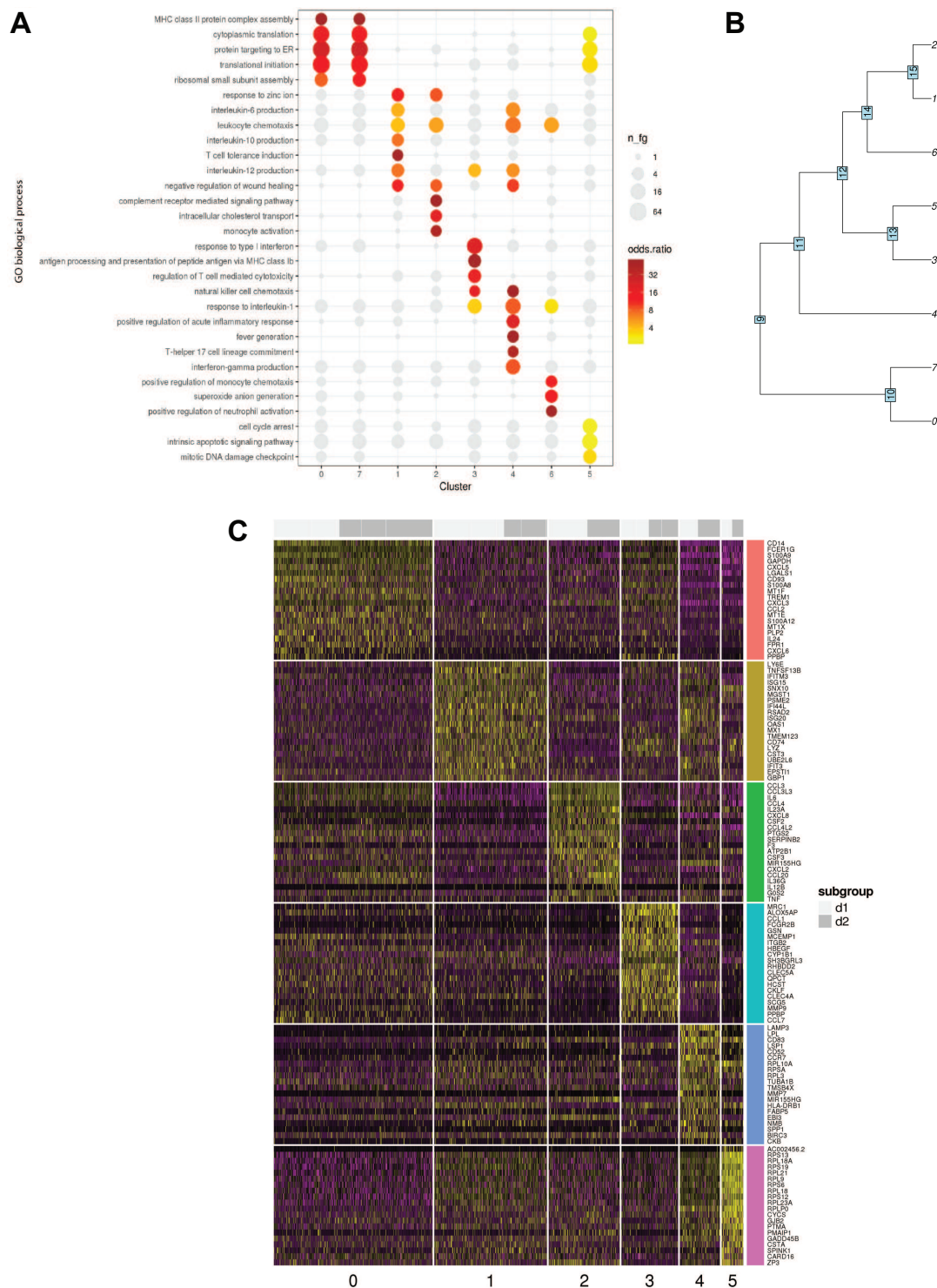

Supplement: Supplementary data [file gutjnl-2020-321731supp010.pdf]

Supplementary Figure 7

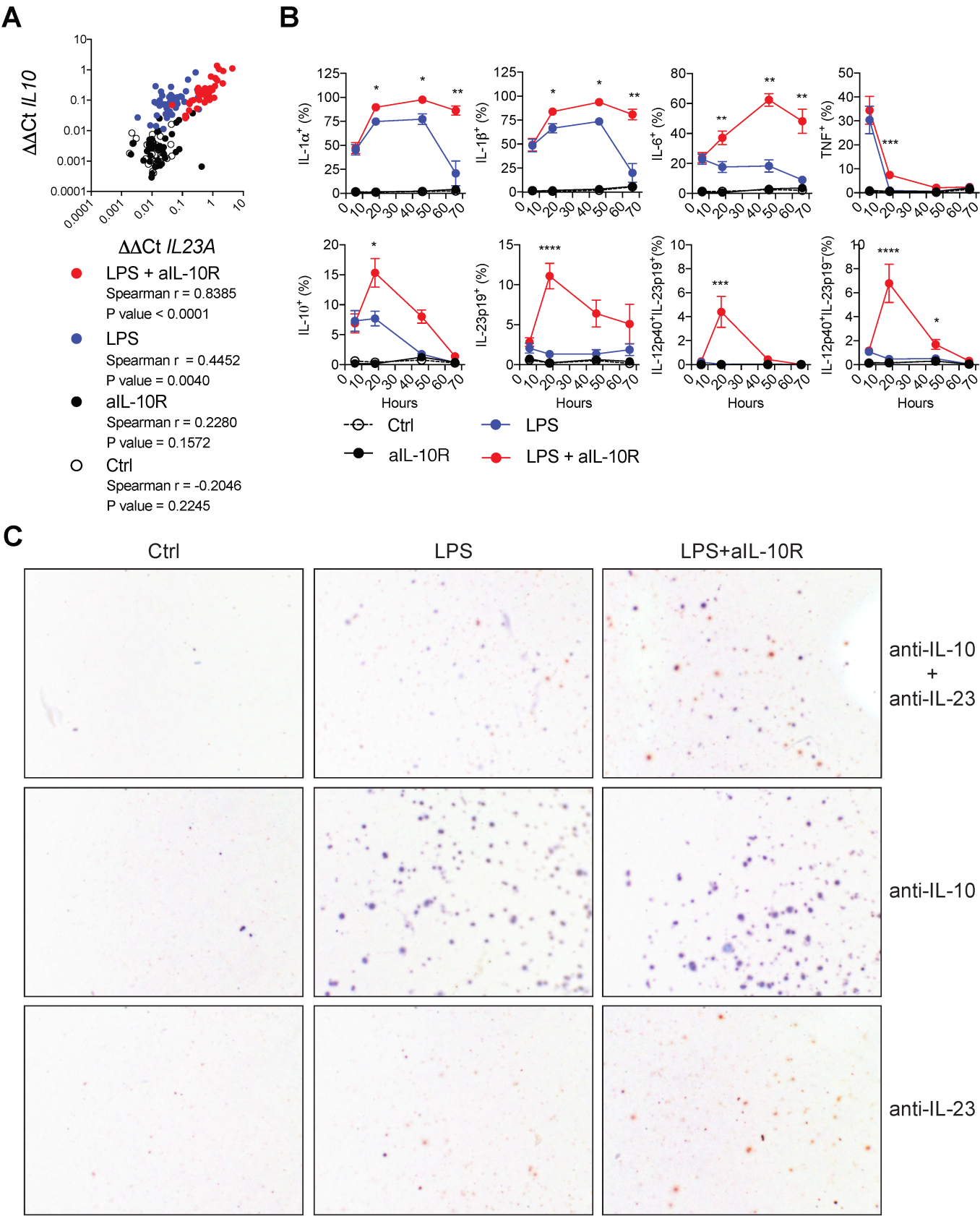

Supplement: Supplementary data [file gutjnl-2020-321731supp011.pdf]

## Supplementary Figure 8

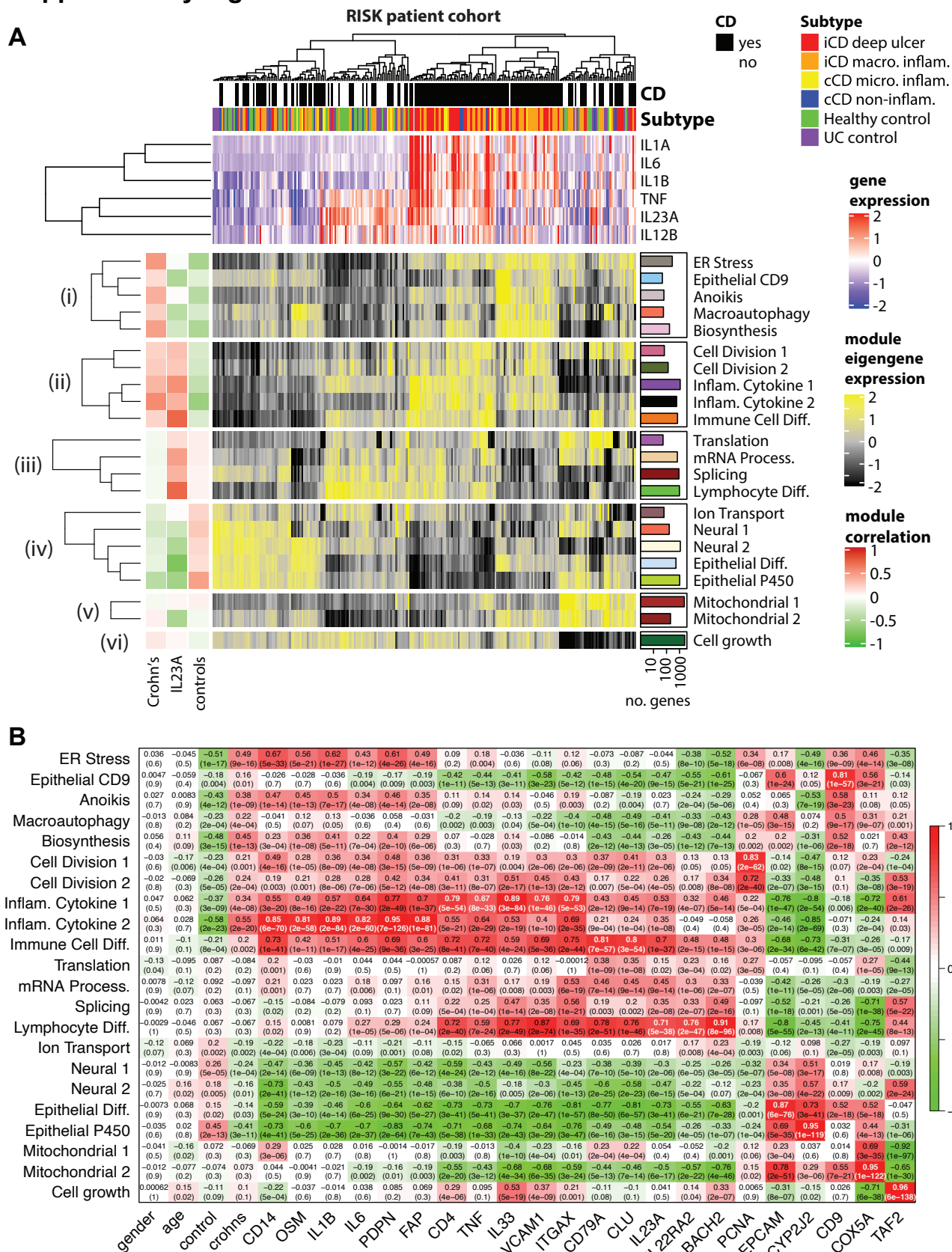

Supplement: Supplementary data [file gutjnl-2020-321731supp012.pdf]

Supplementary Figure 9

A

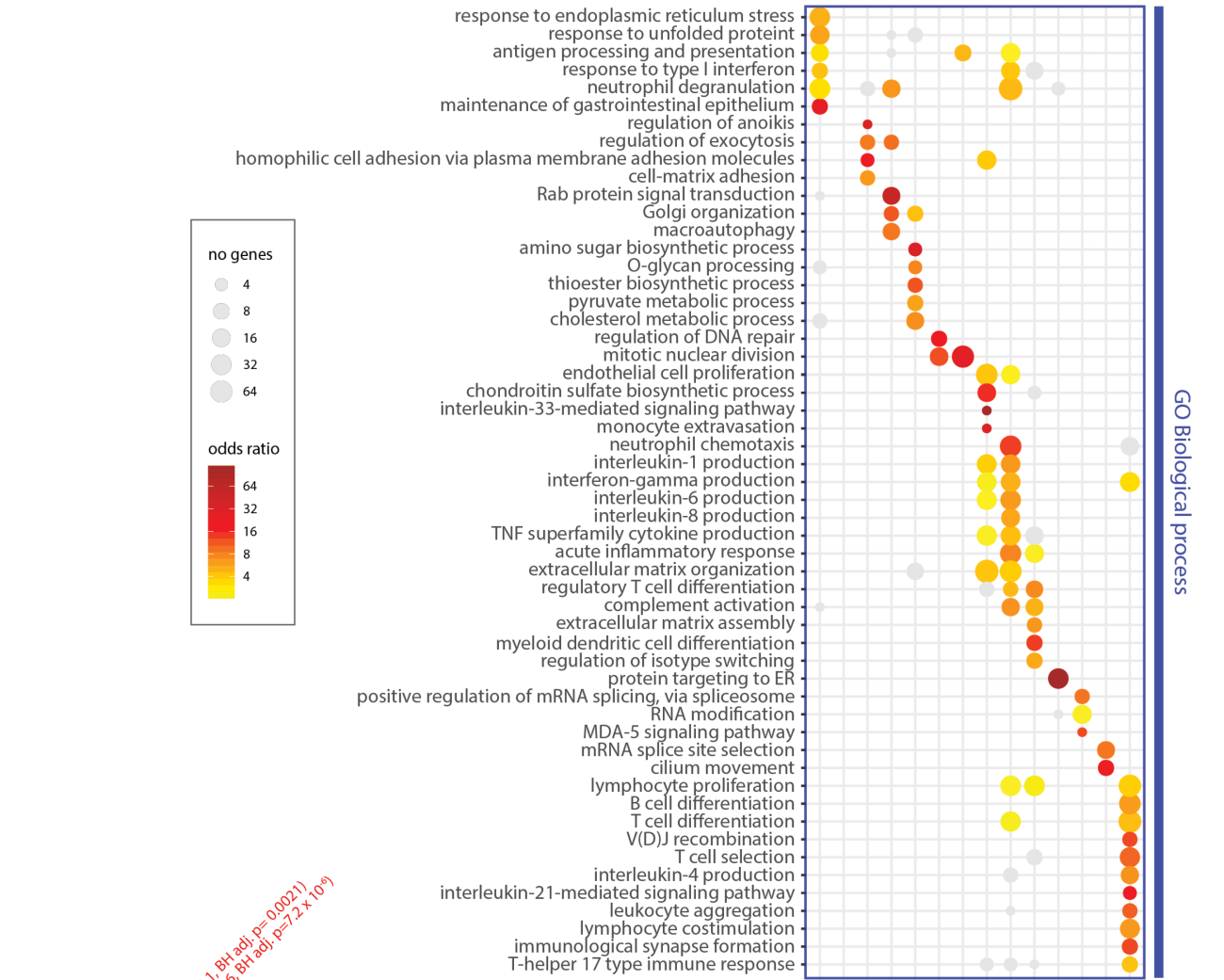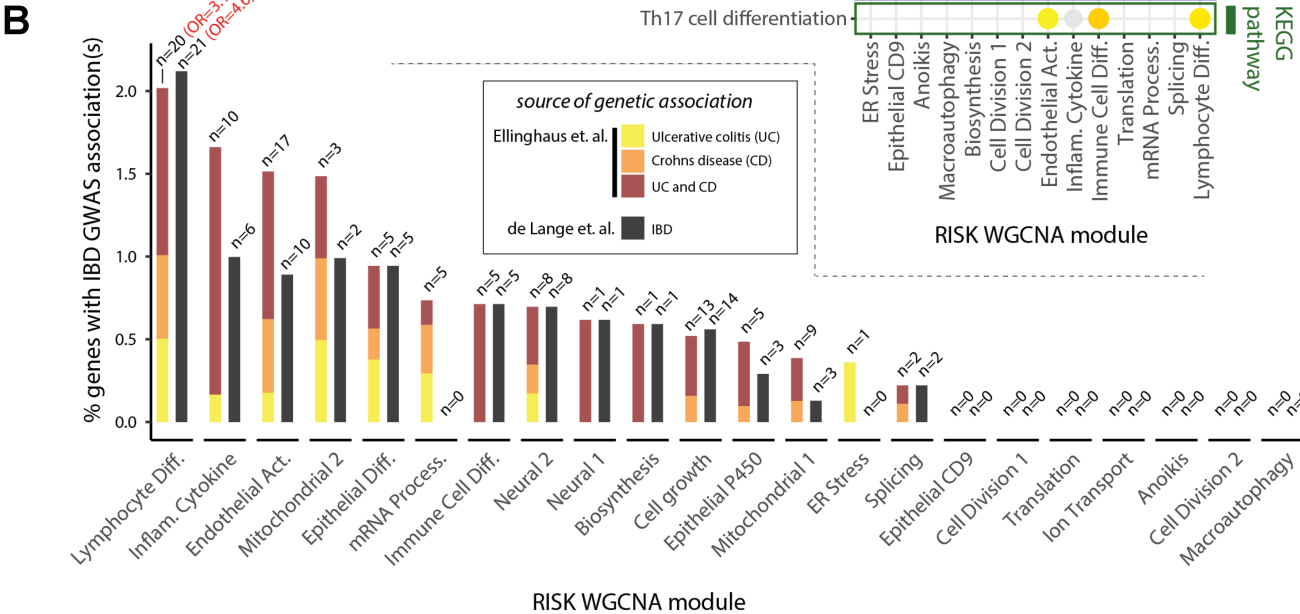

Supplement: Supplementary data [file gutjnl-2020-321731supp014.pdf]

## Supplementary Figure 10

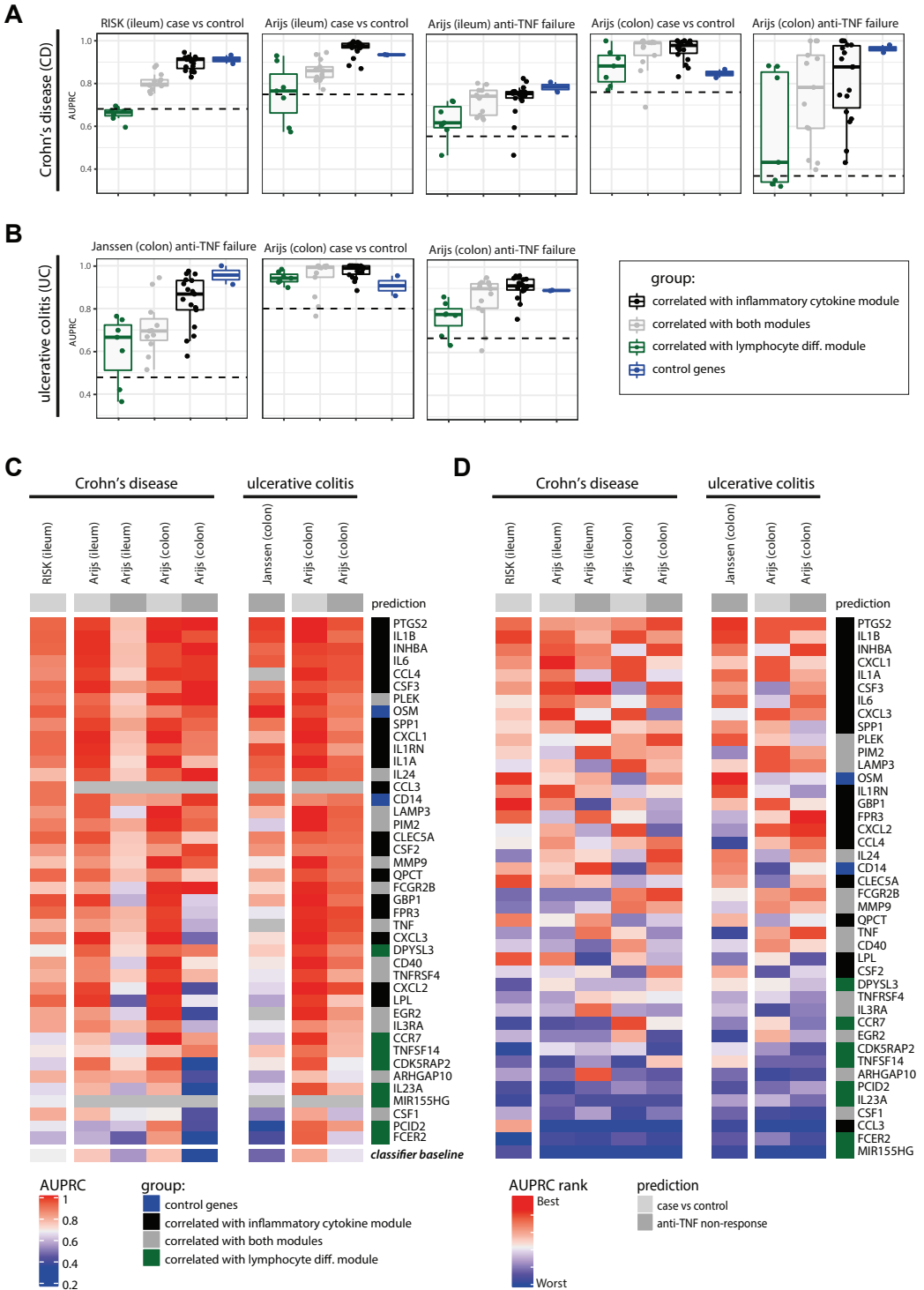

Supplement: Supplementary data [file gutjnl-2020-321731supp016.pdf]

Supplementary Figure 11

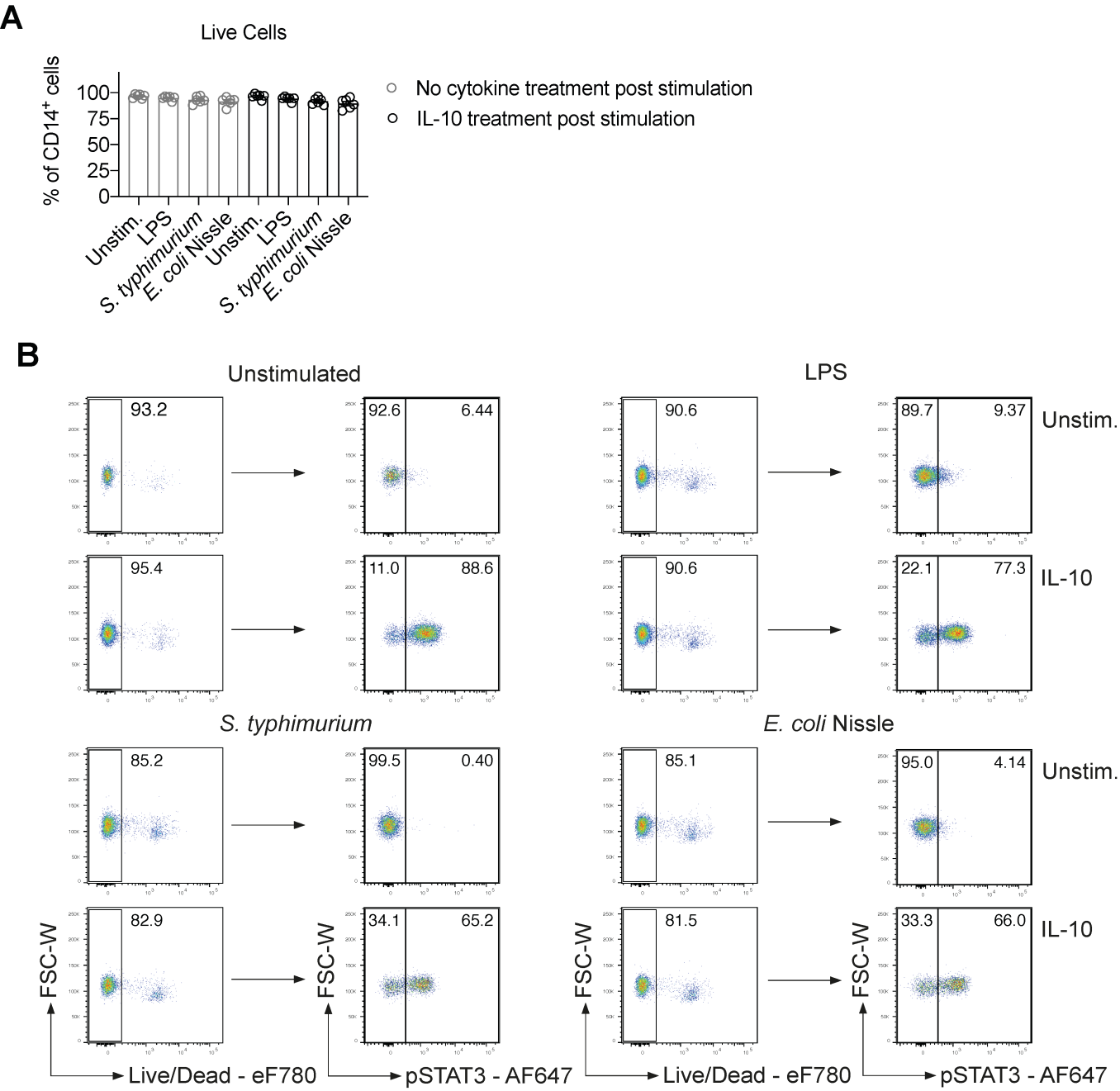

Supplement: Supplementary data [file gutjnl-2020-321731supp017.pdf]

Supplementary Figure 12

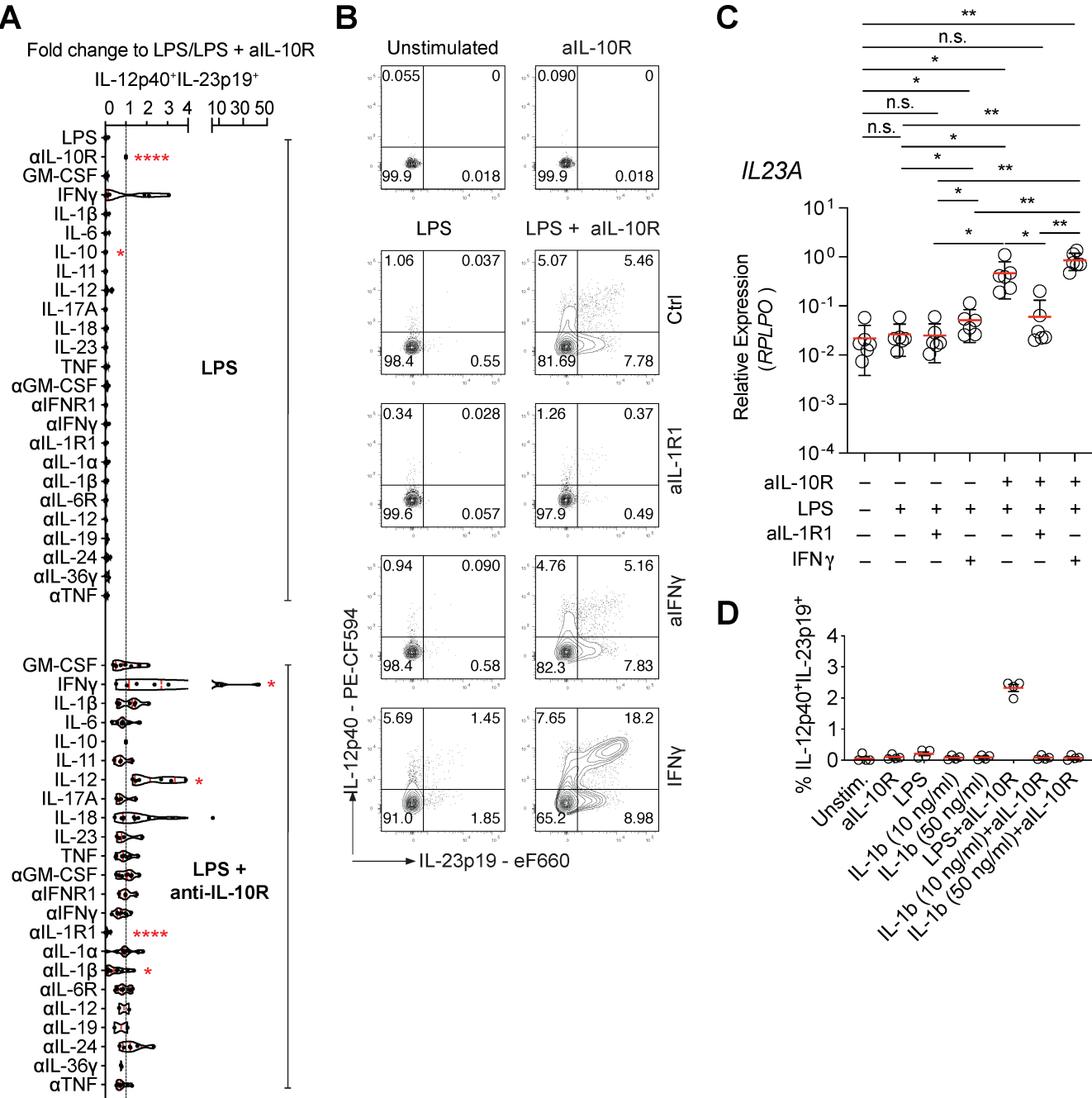

Supplement: Supplementary data [file gutjnl-2020-321731supp018.pdf]

Supplementary Figure 13

A

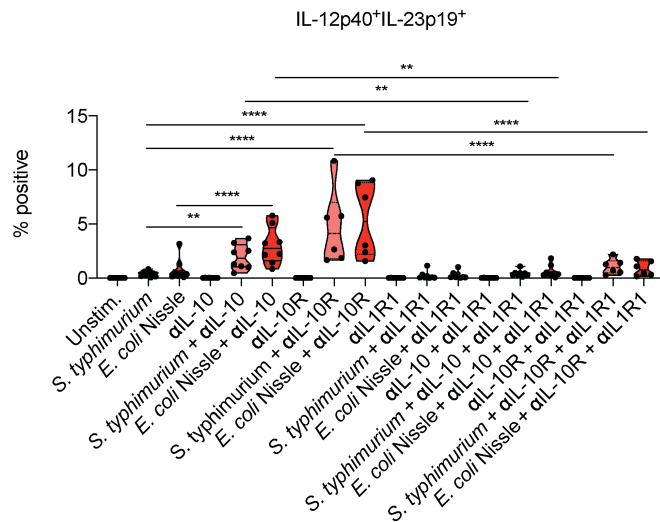

B

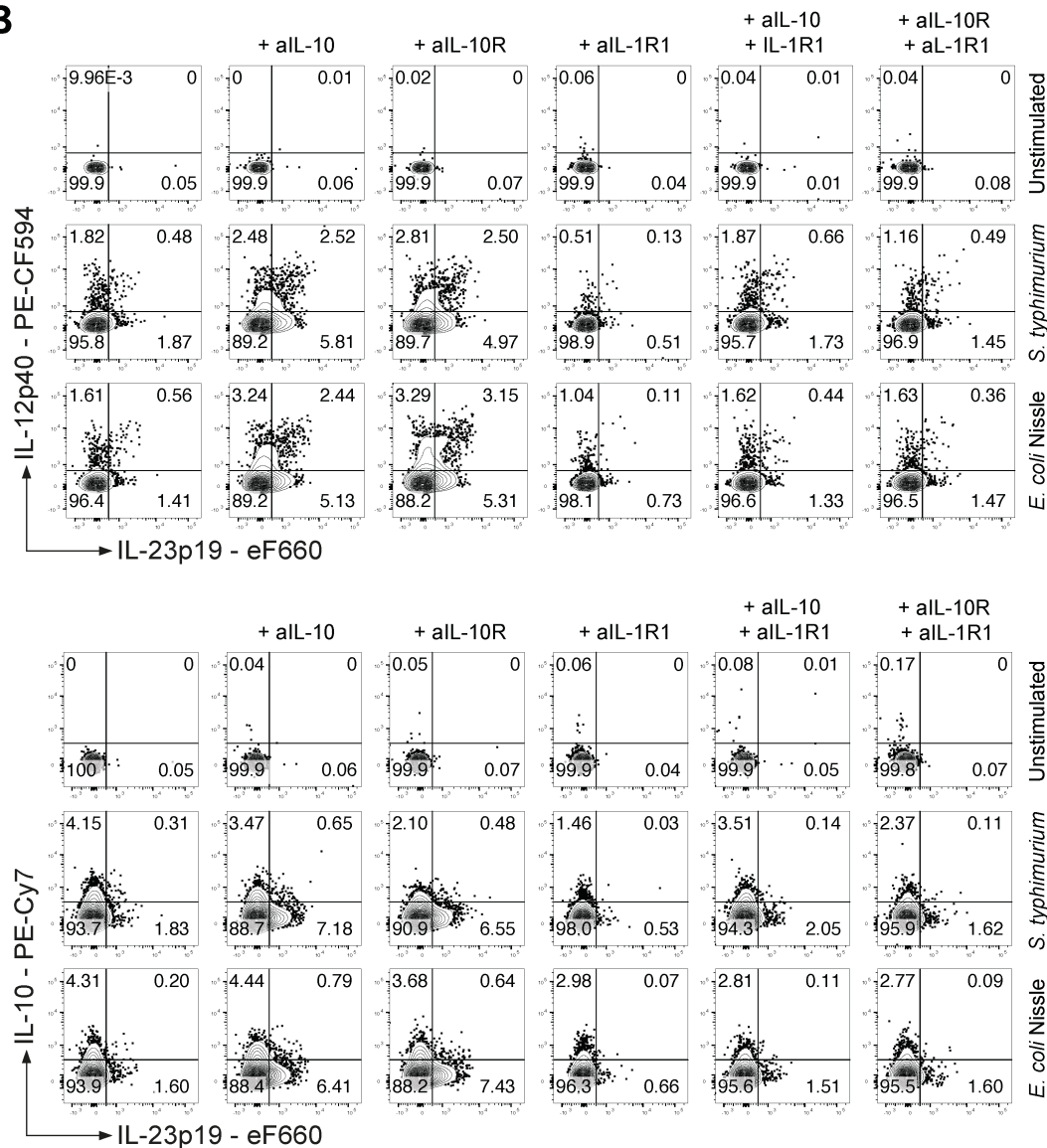

Supplement: Supplementary data [file gutjnl-2020-321731supp019.pdf]

## Graphical Abstract

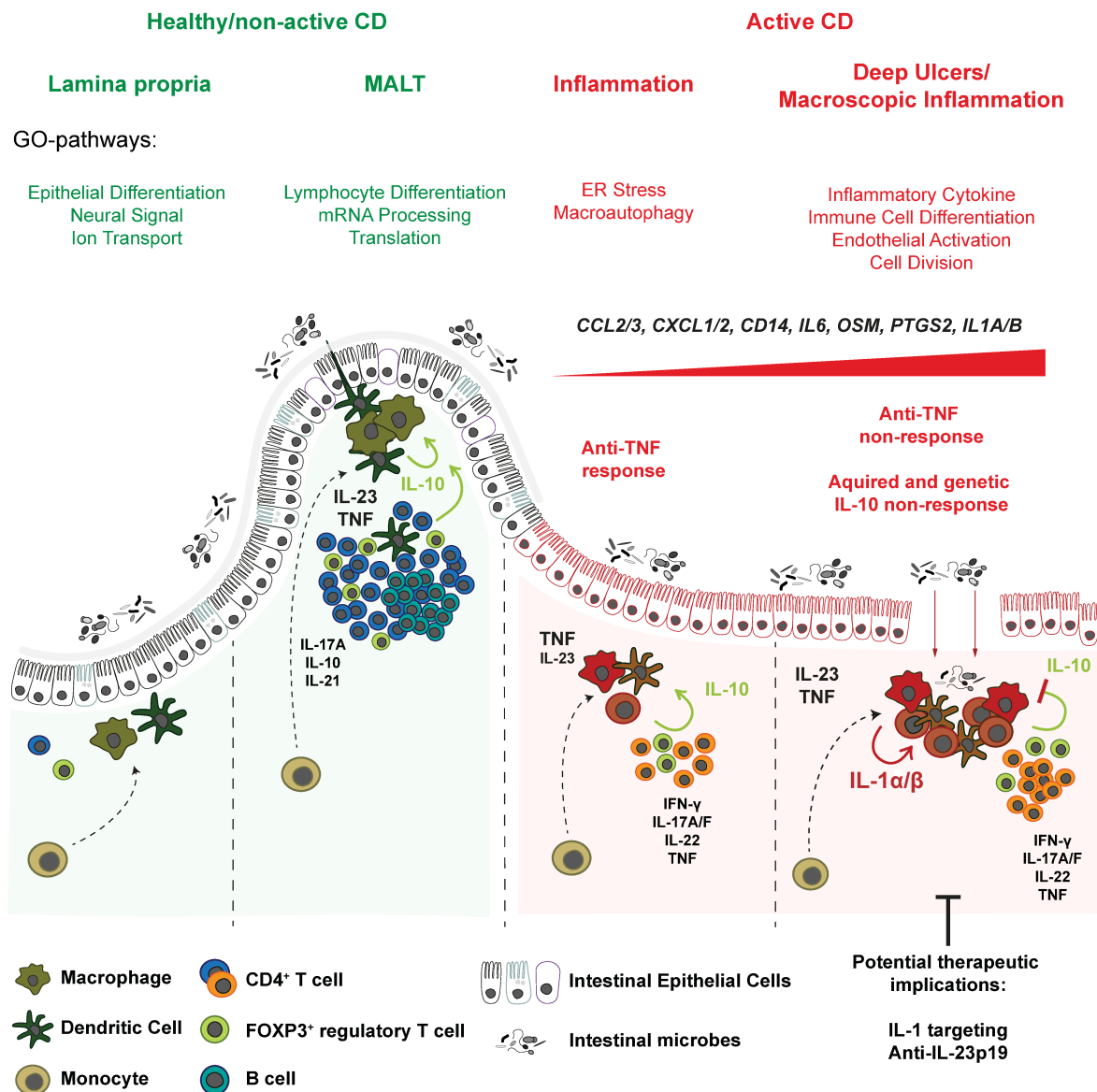

Supplement: Supplementary data [file gutjnl-2020-321731supp020.pdf]
