# Supplementary material for: Deconvolution of monocyte responses in inflammatory bowel disease reveals an IL-1 cytokine network that regulates IL-23 in genetic and acquired IL-10 resistance
Source: Gut. 2020 Oct 9;70(6):1023–36. doi: 10.1136/gutjnl-2020-321731 (PMC8108288; doi:10.1136/gutjnl-2020-321731)
Supplement: Supplementary data [file gutjnl-2020-321731supp003.pdf]

Supplementary Table 1

	Total					Crohn's disease					Ulcerative colitis					IBD unclassified		
**Number of patients (n)**	41					26					13					2		
	**Mean**	**SD**	**Min**	**Max**		**Mean**	**SD**	**Min**	**Max**		**Mean**	**SD**	**Min**	**Max**		**IBDu 1**	**IBDu 2**	
**Age (Years)**	42.3	17.3	18.0	85.0		37.6	15.3	18.0	71.0		51.2	17.5	27.0	85.0		65.0	25.0	
**Age at diagnosis (Years)**	26.0	14.9	9.0	78.0		19.4	8.2	9.0	38.0		37.3	15.4	19.0	78.0		61.0	11.0	
**Gender (Female/Male) (%)**	51.2	48.8				46.2	53.8				61.5	38.5				50.0	50.0	
**Montreal UC (E1/E2/E3) (%)**											0	31	69			0	100	0
**Montreal CD (A1/A2/A3) (%)**						46	54	0										
**Montreal CD (L1/L2/L3/L4) (%)**						8	27	65	19									
**Montreal CD (B1/B2/B3/p) (%)**						38	38	27	42									
**Medication at time of study**																		
**Adalimumab (%)**	17					19					8					0		
**Infliximab (%)**	17					23					8					0		
**Azathioprine/6-MP (%)**	34					35					31					50		
**Methotrexate (%)**	7					4					15					0		
**Remission (%)**	73					77					62					100		
	**Mean**	**SD**	**Min**	**Max**	**NA**	**Mean**	**SD**	**Min**	**Max**	**NA**	**Mean**	**SD**	**Min**	**Max**	**NA**	**IBDu 1**	**IBDu 2**	
**CRP (mg/L) (0.0-5.0)**	2.6	2.9	0.1	13.2	n=3	3.0	2.5	0.1	10.6	n=2	2.1	3.7	0.1	13.2	n=1	2.6	1.1	
**HGB (Female) (g/L) (120-150)**	158	17	136	196		160	17	141	196		157	20	136	184		154.00		
**HGB (Male) (g/L) (130-170)**	155	19	109	196		153	22	109	196		156	13	140	174			152.00	
**RBC (Female) (10*12/L) (3.8-5.8)**	4.8	0.6	3.7	6.0		4.9	0.5	4.1	5.9		4.8	0.8	3.9	6.0		4.6		
**RBC (Male) (10*12/L) (4.5-6.5)**	4.7	0.6	3.1	5.9		4.7	0.7	3.1	5.9		4.6	0.6	3.7	5.3			4.4	
**HCT (Female) (L/L) (0.36-0.46)**	0.46	0.06	0.39	0.57		0.46	0.05	0.40	0.56		0.47	0.06	0.39	0.57		0.44		
**HCT (Male) (L/L) (0.40-0.50)**	0.45	0.06	0.34	0.59		0.45	0.07	0.34	0.59		0.45	0.04	0.40	0.50			0.42	
**MCV (fl) (83 - 101)**	96	6	81	110		95	6	81	110		98	5	93	107		97	98	
**WBC (10*9/L) (4.0-11.0)**	6.6	2.1	3.1	12.8		6.6	2.2	3.1	12.8		6.5	1.9	3.8	10.0		5.6	7.4	
**NEU (10*9/L) (2.0 -7.0)**	4.2	1.6	2.0	8.7		4.3	1.7	2.0	8.7		4.2	1.4	2.3	6.8		2.8	4.8	
**LYM (10*9/L) (1.0 -4.0)**	1.6	0.7	0.5	3.8		1.5	0.6	0.5	2.8		1.8	0.8	0.8	3.8		1.9	1.9	
**MON (10*9/L) (0.2 - 1.0)**	0.5	0.3	0.1	1.3		0.6	0.3	0.1	1.3		0.5	0.2	0.2	0.8		0.7	0.5	
**EOS (10*9/L) (0.02-0.5)**	0.2	0.2	0.0	1.2		0.2	0.2	0.0	1.2		0.2	0.1	0.1	0.3		0.1	0.1	
**BAS (10*9/L) (0.02-0.1)**	0.1	0.1	0.0	0.7		0.1	0.0	0.0	0.2		0.1	0.2	0.0	0.7		0.0	0.1	
**PLT (10*9/L) (150-400)**	219	79	77	450		215	87	77	450		226	67	91	376		159	270	

***Montreal CD:** The sum of percentages of L and B classification can exceed 100 percent since disease classes and modifier were counted individually.

***CRP:** For a total number of 3 patients with IBD no CRP measurements were obtained in a 2 weeks time window in respect to the date of blood sampling.
