# Supplementary material for: Deconvolution of monocyte responses in inflammatory bowel disease reveals an IL-1 cytokine network that regulates IL-23 in genetic and acquired IL-10 resistance
Source: Gut. 2020 Oct 9;70(6):1023–36. doi: 10.1136/gutjnl-2020-321731 (PMC8108288; doi:10.1136/gutjnl-2020-321731)

Supplementary Figure 5

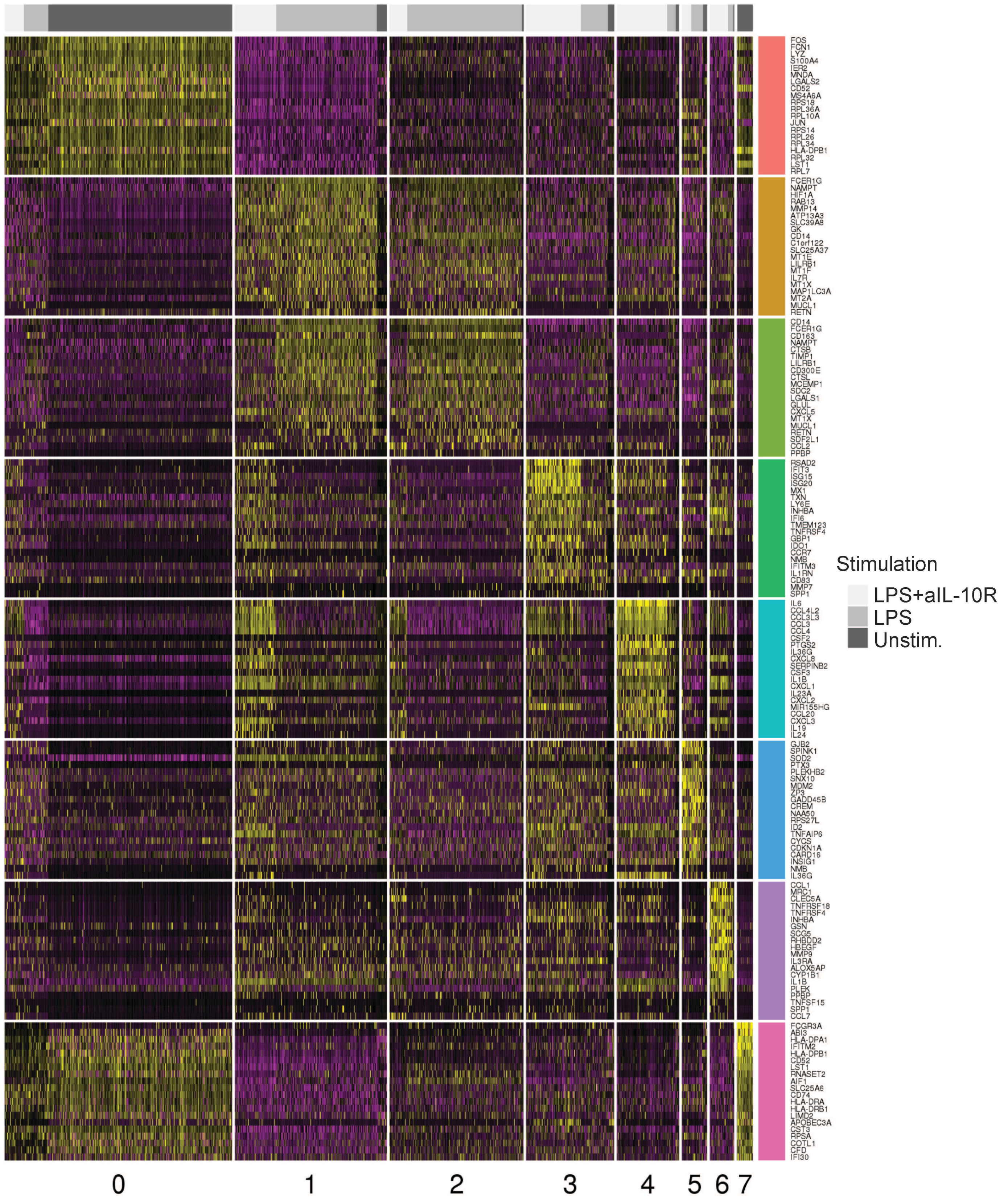

Supplement: Supplementary data [file gutjnl-2020-321731supp009.pdf]
